# The association between insulin resistance assessed by estimated glucose disposal rate and stroke prevalence and mortality in non-diabetic people: evidence from two prospective cohorts

**DOI:** 10.1186/s13098-025-01956-6

**Published:** 2025-10-10

**Authors:** Dandan Li, Yan Guo, Fanfan Cui, Yajing Cheng, Shufang Yan, Tong Yu, Xuejiao Li, Zhanju Liu, Hongwei Jiang

**Affiliations:** 1 The First Affiliated Hospital and Clinical Medicine College, Henan University of Science and Technology, 636 Guanlin Road, Luoyang, Henan Province China; 2 Huaihe Hospital of Henan University, Kaifeng, Henan Province China; 3 Shanghai Tenth People’s Hospital, Tongji University School of Medicine, 301 Yanchang Road, Shanghai, China

**Keywords:** Stroke, Insulin resistance, Estimated glucose disposal rate, NHANES, CHARLS, Mortality, Non-Diabetic people

## Abstract

**Background:**

The estimated glucose disposal rate (eGDR), serving as a measure of insulin resistance (IR), provides a simpler and more accessible method for assessing insulin sensitivity. However, its association with stroke and mortality in non-diabetic patients remains to be fully clarified.

**Methods:**

Data from the National Health and Nutrition Examination Survey (NHANES) Study (2003–2014, *n* = 11,063, age ≥ 45) were examined. Participants with diabetes, coronary heart disease (CHD), or missing key data were excluded. eGDR was calculated based on waist circumference, hypertension status, and glycated hemoglobin (HbA1c). The primary outcomes were stroke prevalence and all-cause, cardiovascular, and cerebrovascular disease mortality. For stroke outcomes, a cross-sectional analysis was conducted and multivariate logistic regression was employed for assessment; whereas for all mortality outcomes, longitudinal analysis was performed using multivariate Cox proportional hazards models. The association between eGDR and these outcomes was investigated using Kaplan-Meier survival analysis, Cox proportional hazards regression models, restricted cubic splines (RCS), and mediation analysis, adjusted for demographic and clinical variables. An additional cohort of 6,873 participants from the China Longitudinal Health and Aging Study (CHARLS) was applied to evaluate the association further.

**Results:**

Between 2003 and 2014, the NHANES study documented 380 stroke cases. A higher eGDR showed a significant linked to lower stroke prevalence (OR 0.44, 95% CI: 0.31–0.62, *P* < 0.001), as well as reduced all-cause mortality (HR 0.78, 95% CI: 0.68–0.89), CVD mortality (HR 0.70, 95% CI: 0.53–0.94) and cerebrovascular diseases mortality (HR 0.54, 95% CI: 0.31–0.94) over a median 117.6 months. Each one-unit increase in eGDR was associated with a 15% decrease in stroke prevalence (OR 0.85, 95% CI 0.79–0.9). The protective impact of a higher eGDR against stroke was consistently observed across most subgroups, but significantly stronger in individuals < 60 years. CHARLS demonstrated the research findings.

**Conclusions:**

Elevated eGDR levels showed an independent correlation with a significantly reduced prevalence of stroke and decreased risks of mortality in non-diabetic adults.

**Graphical abstract:**

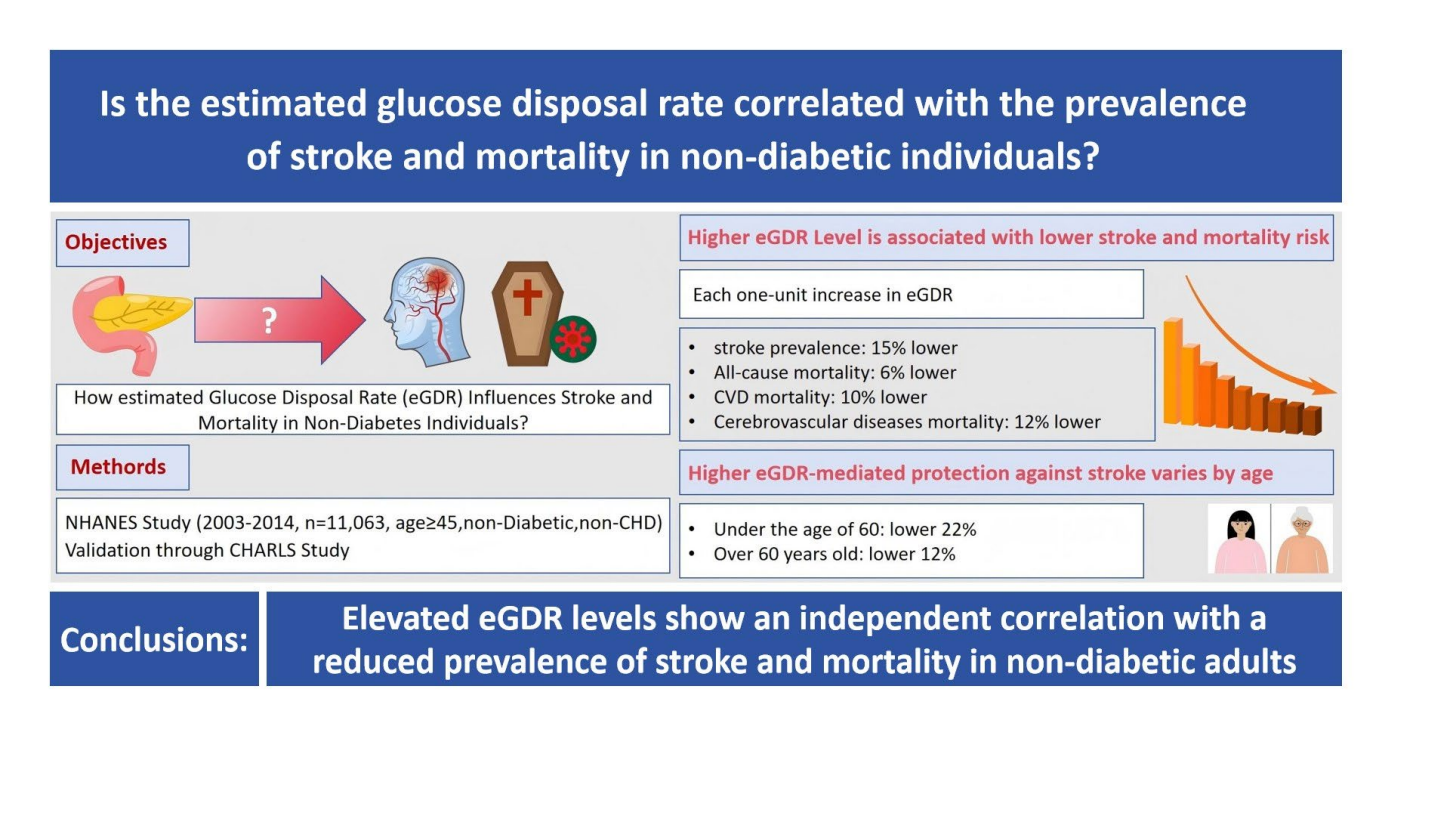

**Supplementary Information:**

The online version contains supplementary material available at 10.1186/s13098-025-01956-6.

## Background

Stroke ranks as the second most common cause of death from non-communicable diseases among adults in the Global Burden of Disease (GBD) study, second only to ischemic heart disease [1, 2]. It is also the fourth most common cause of disability, resulting in 1.605 billion disability-adjusted life years (DALYs) [3]. Over the past three decades, stroke-related deaths and long-term disabilities have nearly doubled. Notably, the incidence of stroke is rising at a faster pace in low- and middle-income countries than in high-income countries [1, 4]. Therefore, it is necessary to propose practical solutions based on evidence-based medical evidence to alleviate the worldwide impact of stroke.

Insulin resistance (IR) describes the diminished ability of target tissues to respond effectively to insulin. It is broadly acknowledged as a significant factor in the development of cardiovascular diseases (CVD) and increased mortality risk [5, 6]. The hyperinsulinemic-euglycemic glucose clamp (HEGC) is the most reliable method for assessing IR [7]. However, the time-consuming and resource-intensive nature of HEGC detection has hindered its implementation.

Recently, some simpler methods have been proposed to assess IR [8, 9]. The estimated glucose disposal rate (eGDR) elucidates the interconnection among obesity, hypertension, and glucose metabolism by incorporating routinely utilized clinical assessment indicators, including waist circumference (WC), glycated hemoglobin A1c (HbA1c), and the presence of hypertension [10, 11]. Prior research has shown that elevated eGDR levels indicate higher insulin sensitivity and are negatively correlated with the risk of stroke in diabetic patients [12, 13]. However, there are still no studies specifically targeting non-diabetic patients. To bridge this research gap, we selected participants from the “National Health and Nutrition Examination Survey” (NHANES) to evaluate the correlation between eGDR and stroke prevalence and mortality. Additionally, we used the data from the “China Longitudinal Health and Aging Study” (CHARLS) as a second independent dataset to further clarify this association.

## Methods

### Participant cohort

NHANES is a comprehensive, multi-phase program conducted to assess the US civilian population’s overall national and regional health and nutrition status. Survey data collection involved a standardized questionnaire that captured information on demographics, socioeconomic conditions, and previous medical history. Blood samples were collected at the mobile testing site, stored at −20 °C, and transported to central laboratories for further analysis. From 2003 to 2014, over 60,000 participants were recruited for the NHANES study. To match the age distribution with that of the CHARLS cohort, the survey did not include individuals under 45 and those in pregnancy (*n* = 42,050). Subsequently, we conducted a sensitivity analysis on patients aged 20 years or older. Exclusion criteria included: missing eGDR data (*n* = 2,646); having been diagnosed with diabetes, or meeting the diagnostic criteria for diabetes defined as a Fasting Plasma Glucose(FPG)≥7.0 mmol/L or HbA1c ≥ 6.5% (*n* = 3,983). To further elucidate the influence of eGDR on stroke prevalence, we excluded participants who had already been diagnosed with coronary heart disease (CHD) (*n* = 1,327). Patients with missing mortality data were also deleted (*n* = 18).

To further clarify the research results obtained from the NHANES, we further analyzed the baseline and follow-up information of the “China Health and Retirement Longitudinal Study” (CHARLS) [14]. This research represented a longitudinal survey with national representation, focusing on individuals 45 or older in China, with a follow-up survey conducted every two years. The initial assessment took place from June 2011 to March 2012. This study included 6,873 participants, while the remaining participants were excluded for the above reasons.

### Determination of exposure factors and outcome measures

Baseline eGDR was used as the exposure variable. The equation for determining eGDR was provided: eGDR (mg/kg/min) = 21.158 - (0.09*waist circumference) - (3.407 *hypertension) - (0.551*HbA1c) [waist circumference (cm), hypertension (yes = 1/no = 0), and HbA1c (%)] [15]. Hypertension was diagnosed as systolic blood pressure (SBP) ≥ 140 mmHg, diastolic blood pressure (DBP) ≥ 90 mmHg, current use of antihypertensive medication, or a self-reported diagnosis of hypertension on at least two occasions. The study population was stratified into three groups based on the eGDR tertile levels. The group with the lowest eGDR level represented the most severe IR. The primary outcome measure of interest was stroke or mortality. Diagnosed stroke cases were identified through self-reported information provided by the participants.

### Mortality assessment

In the NHANES study, the survival status of participants was determined by matching individual IDs with national death index records. Follow-up time was calculated from the Mobile Examination Center assessment date to the earlier date of death or December 31, 2019. The CHARLS dataset provided mortality data availability up to Wave 5, covering information through 2020. CVD mortality and Cerebrovascular disease mortality were defined based on ICD-10 codes.

### Covariates of interest

To ensure research reliability, we included several variables in the analysis: Age, Sex, Education Levels, Marital Status, Smoking and Alcohol Consumption, Race (NHANES only), and history of chronic diseases, including Hypertension, Hyperlipemia, and the administration of antihypertensive and lipid-lowering drugs. Economic status (NHANES only) was assessed using the family income-to-poverty ratio (PIR). Sex (male/female) was self-reported by participants in both NHANES and CHARLS. All variables were collected via standardized household interviews.

### Statistical analysis

Continuous variables with normal distribution are summarized as mean ± standard deviation (SD), non-normally distributed variables as median (interquartile range, IQR), and categorical data as counts and percentages. Group comparisons employed Chi-square tests for categorical variables, one-way ANOVA with Levene’s test for normally distributed continuous variables (assessing variance homogeneity), and Kruskal-Wallis tests for non-normally distributed continuous variables.

Stroke outcomes were analyzed cross-sectionally using multivariate logistic regression, while all mortality outcomes were assessed longitudinally with multivariate Cox proportional hazards models. eGDR was analyzed continuously (per 1-unit increment) and categorically (tertiles; Q1 as reference). Three models were constructed: Model 1 (crude), Model 2 (adjusted for age, sex, education, smoking, alcohol, marital status, race [NHANES only], PIR [NHANES only]), and Model 3 (Model 2 covariates plus BMI, LDL-C, TC, TG, lipid-lowering drugs, hs-CRP [CHARLS only], UA [CHARLS only]). The proportional hazards assumption was verified through Schoenfeld residual tests, confirming no significant violations (*P* > 0.05). Kaplan-Meier curves depicted cumulative mortality incidence, with group differences assessed by the log-rank test. Restricted cubic splines (RCS) and multivariable Cox regression explored potential linear/nonlinear relationships between baseline eGDR and outcomes.

Stratified subgroup analyses examined effect modification by sex, age (< 60, ≥ 60), BMI (< 25, 25–30, ≥ 30), smoking status, alcohol consumption, and race. A DerSimonian-Laird random-effects meta-analysis synthesized log-transformed hazard ratios (HRs) and 95% confidence intervals (CIs) from Model 3 across cohorts to assess eGDR and all-cause mortality associations.

Mediation analysis (Valeri and VanderWeele method) [16] evaluated whether lipids (TC, LDL-C, HDL-C, TG) mediated the established eGDR-stroke/mortality associations. Sensitivity analyses included: (1) excluding participants with critical missing covariates, (2) including NHANES participants aged ≥ 20 years. Fine-Gray subdistribution hazard models addressed competing risks. Missing data were managed via list-wise deletion and multiple imputation (5 replications).

Analyses used R (v4.4.1) and Free Statistics Analysis Platform (v2.0). Statistical significance was defined as a 95% CI excluding null or a two-sided *P*-value < 0.05.

## Results

### Baseline features

From 2003 to 2014, the NHANES datasets included 19,037 adults aged 45 years or older, comprising both males and non-pregnant females. After excluding 7,974 individuals due to missing data required for eGDR and mortality, and those already diagnosed with diabetes and CHD, the final analytic sample consisted of 11,063 participants (Figure S1). The cohort’s median age was 60.8(SD = 11.1), with 5,203 males accounting for 47.0% of the sample. The number of patients diagnosed with stroke was 380, accounting for 3.4% of the entire population. The average eGDR was 7.7 mg/kg/min (SD = 2.3). Patients with greater eGDR levels tended to be females, younger, non-smokers, and non-drinkers, and exhibited relatively high HDL-C levels along with low BMI, insulin, and TG levels (Table 1).

**Table 1:** Baseline characteristics of 11,063 participants by tertile of eGDR in the NHANES study

Variables	Total (*n* = 11063)	eGDR(mg/kg/min)			^a^*P* -value
		Q1(< 6.38) (*n* = 3687)	Q2(6.38–9.14)) (*n* = 3687)	Q3(> 9.14) (*n* = 3689)	
Age, y	60.8 ± 11.1	63.7 ± 10.9	61.5 ± 11.3	57.4 ± 10.2	< 0.001
Sex, n (%)					< 0.001
Male	5203 (47.0)	1847 (50.1)	1789 (48.5)	1567 (42.5)	
Female	5860 (53.0)	1840 (49.9)	1898 (51.5)	2122 (57.5)	
Race, n (%)					< 0.001
Non-Hispanic White	5795 (52.4)	1934 (52.5)	1983 (53.8)	1878 (50.9)	
Non-Hispanic Black	2120 (19.2)	879 (23.8)	691 (18.7)	550 (14.9)	
Hispanic	1490 (13.5)	480 (13)	506 (13.7)	504 (13.7)	
Non-Hispanic Asian	874 (7.9)	242 (6.6)	287 (7.8)	345 (9.4)	
Other Race	784 (7.1)	152 (4.1)	220 (6)	412 (11.2)	
Education, n (%)					< 0.001
Less than 9th grade	1408 (12.7)	503 (13.6)	473 (12.8)	432 (11.7)	
9-11th grade	1502 (13.6)	549 (14.9)	523 (14.2)	430 (11.7)	
High school	2627 (23.7)	965 (26.2)	878 (23.8)	784 (21.3)	
College or above	2914 (26.3)	962 (26.1)	998 (27.1)	954 (25.9)	
None of the above	2612 (23.6)	708 (19.2)	815 (22.1)	1089 (29.5)	
Smoking status, n (%)					< 0.001
Never	5622 (50.8)	1887 (51.2)	1789 (48.5)	1946 (52.8)	
Former	3656 (33.0)	1353 (36.7)	1257 (34.1)	1046 (28.4)	
Current	1785 (16.1)	447 (12.1)	641 (17.4)	697 (18.9)	
Alcohol consumption, n (%)					< 0.001
No	9526 (86.1)	3110 (84.4)	3166 (85.9)	3250 (88.1)	
Yes	1537 (13.9)	577 (15.6)	521 (14.1)	439 (11.9)	
Marital status, n (%)					< 0.001
Not married nor living with a partner	4121 (37.3)	1443 (39.1)	1395 (37.8)	1283 (34.8)	
Married or living with a partner	6942 (62.7)	2244 (60.9)	2292 (62.2)	2406 (65.2)	
PIR	2.8 ± 1.6	2.7 ± 1.6	2.8 ± 1.6	3.0 ± 1.7	< 0.001
BMI, kg/m2	28.3 ± 5.9	31.7 ± 6.2	28.4 ± 5.5	24.7 ± 3.4	< 0.001
HbA1c, %	5.5 ± 0.4	5.6 ± 0.4	5.6 ± 0.4	5.4 ± 0.3	< 0.001
FBG, mmol/L	5.6 ± 0.6	5.8 ± 0.6	5.6 ± 0.5	5.4 ± 0.5	< 0.001
Ins, uU/mL	11.7 ± 9.2	15.1 ± 10.7	11.8 ± 9.0	8.2 ± 6.1	< 0.001
Cho, mg/dl	206.2 ± 40.2	205.8 ± 41.3	206.1 ± 39.9	206.8 ± 39.4	0.556
LDL, mg/dl	115.5 ± 35.4	115.3 ± 35.3	115.5 ± 34.9	115.7 ± 36.1	0.882
TG, mg/dl	121.0 (67.0, 228.0)	139.0 (76.0, 253.0)	122.0 (68.0, 231.0)	105.0 (61.0,198.0)	< 0.001
HDL, mg/dl	55.9 ± 16.8	52.5 ± 15.3	55.5 ± 17.1	59.8 ± 17.0	< 0.001
eGDR, mg/kg/min	7.7 ± 2.3	5.0 ± 1.0	7.8 ± 0.8	10.2 ± 0.7	< 0.001
SBP, mmHg	129.0 ± 19.7	139.1 ± 20.6	129.9 ± 19.4	117.9 ± 11.5	< 0.001
DBP, mmHg	71.4 ± 13.3	73.4 ± 15.5	71.2 ± 13.5	69.5 ± 10.2	< 0.001
hypertension, n (%)					< 0.001
No	6061 (54.8)	123 (3.3)	2249 (61)	3689 (100)	
Yes	5002 (45.2)	3564 (96.7)	1438 (39)	0 (0)	
Antihypertensive drugs, n (%)					< 0.001
No	8112 (73.3)	1464 (39.7)	2959 (80.3)	3689 (100)	
Yes	2951 (26.7)	2223 (60.3)	728 (19.7)	0 (0)	
hyperlipemia, n (%)					< 0.001
No	6692 (60.5)	1823 (49.4)	2278 (61.8)	2591 (70.2)	
Yes	4371 (39.5)	1864 (50.6)	1409 (38.2)	1098 (29.8)	
Lipid-lowering drugs, n (%)					< 0.001
No	9289 (84.0)	2722 (73.8)	3162 (85.8)	3405 (92.3)	
Yes	1774 (16.0)	965 (26.2)	525 (14.2)	284 (7.7)	
Stroke, n (%)					< 0.001
No	10,683 (96.6)	3490 (94.7)	3569 (96.8)	3624 (98.2)	
Yes	380 (3.4)	197 (5.3)	118 (3.2)	65 (1.8)	

Values for categorical variables were presented as count (%), mean ± SD. TG variable was presented as the median and interquartile range (IQR). Abbreviations: BMI, body mass index; Cho, Cholesterol; TG, triglyceride; DBP, diastolic blood pressure; eGDR, estimated glucose disposal rate; FBG, Fasting blood glucose; HbA1c, hemoglobin A1c; HDL-C, high-density lipoprotein cholesterol; LDL-C, low-density lipoprotein cholesterol; PIR, poverty-to-income ratio; SBP: systolic blood pressure

^a^ *P* values derived from χ2 tests (categorical variables) or ANOVA (continuous variables) comparing values across tertiles

### Association between eGDR and prevalence of stroke

The higher eGDR group showed a lower prevalence of stroke after adjustment. The odds ratios (OR) for stroke prevalence were 0.63 (95%CI: 0.49–0.82) in Q2, and 0.44 (95% CI: 0.31–0.62) in Q3(all *P* < 0.001). Each 1-unit increment in eGDR was Linked to a 15% lower stroke prevalence (OR = 0.85, 95% CI: 0.79–0.90, *P* < 0.001) (Table 2). RCS analysis with the fully adjusted analyses showed no evidence of a significant nonlinear association between eGDR levels and stroke prevalence (Figure S2).

**Table 2:** Association between eGDR and stroke prevalence in the NHANES and CHARLS studies

Variable	Event, *n* (%)	Model 1		Model 2		Model 3	
		OR (95%CI)	*P* value	OR (95%CI)	*P* value	OR (95%CI)	*P* value
**NHANES**							
Continuous variable per unit	380 (3.4)	0.83(0.8 ~ 0.87)	< 0.001	0.88 (0.83 ~ 0.92)	< 0.001	0.85 (0.79 ~ 0.9)	< 0.001
Q1(< 6.38)	197 (5.3)	1(Ref)		1(Ref)		1(Ref)	
Q2(6.38–9.14)	118 (3.2)	0.59 (0.46 ~ 0.74)	< 0.001	0.64 (0.5 ~ 0.81)	< 0.001	0.63 (0.49 ~ 0.82)	< 0.001
Q3(> 9.14)	65 (1.8)	0.32 (0.24 ~ 0.42)	< 0.001	0.46 (0.34 ~ 0.62)	< 0.001	0.44 (0.31 ~ 0.62)	< 0.001
Trend test	380 (3.4)	0.57 (0.5 ~ 0.65)	< 0.001	0.67 (0.58 ~ 0.77)	< 0.001	0.66 (0.56 ~ 0.78)	< 0.001
**CHARLS**							
Continuous variable per unit	120 (1.7)	0.78 (0.71 ~ 0.85)	< 0.001	0.8 (0.73 ~ 0.87)	< 0.001	0.8 (0.72 ~ 0.89)	< 0.001
Q1(< 8.30)	69 (3)	1(Ref)		1(Ref)		1(Ref)	
Q2(8.30-10.91)	32 (1.4)	0.46 (0.3 ~ 0.7)	< 0.001	0.5 (0.32 ~ 0.76)	0.001	0.53 (0.34 ~ 0.82)	0.004
Q3(> 10.91)	19 (0.8)	0.27 (0.16 ~ 0.45)	< 0.001	0.31 (0.19 ~ 0.52)	< 0.001	0.34 (0.2 ~ 0.59)	< 0.001
Trend test	120 (1.7)	0.51 (0.39 ~ 0.65)	< 0.001	0.55 (0.43 ~ 0.7)	< 0.001	0.57 (0.44 ~ 0.75)	< 0.001

Model 1: crude. Model 2: age, sex, education, smoking status, alcohol consumption, marital status, race (NHANES only), and PIR (NHANES only). Model 3 is adjusted for model 2 covariates plus BMI, LDL-C, Cho, TG, Lipid-lowering drugs, hs-CRP (CHARLS only), and UA (CHARLS only). Abbreviations: CHARLS, China Health and Retirement Longitudinal Study; NHANES, National Health and Nutrition Examination Survey

### Association between eGDR and mortality

**Fig. 1:**
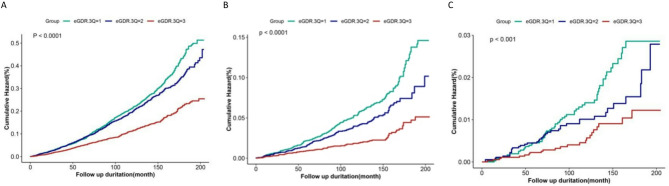
Cumulative incidence of (**A**) all-cause mortality, (**B**) all-cause mortality, and (**C**) cerebrovascular disease mortality according to baseline eGDR tertiles in the NHANES Study. CVD, cardiovascular disease

**Fig. 2:**
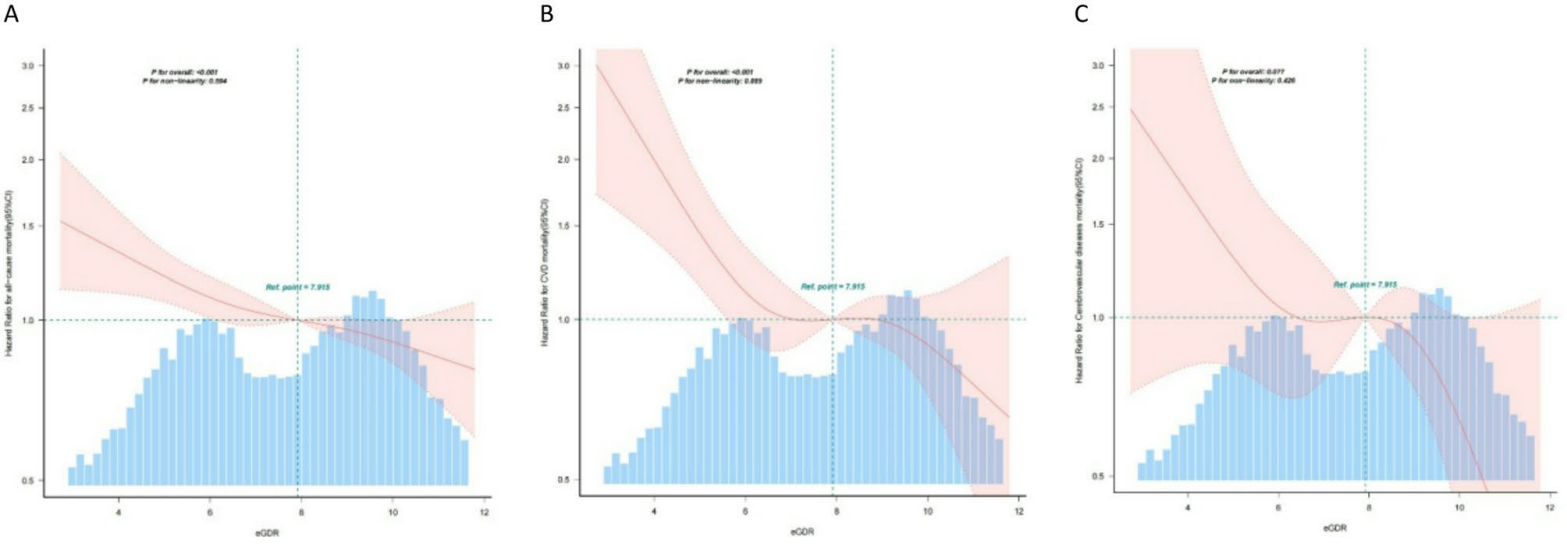
HR and 95% CI for risk of (**A**) all-cause mortality, (**B**) all-cause mortality, and (**C**) cerebrovascular disease mortality associated with eGDR in the NHANES Study. The red solid Line represents the HR modeled using a restricted cubic spline with 4 knots of eGDR. Shaded areas represent 95% CIs for HR. Based on the fully adjusted model (Cox model)

A total of 2,046 deaths were documented over the entire follow-up period, with a median follow-up duration of 117.6 months. The Q3 group demonstrated a markedly lower cumulative incidence of all-cause, CVD, and cerebrovascular disease mortality than the Q1 group (Fig. 1; Table 3). Mortality risk decreased significantly with higher eGDR tertiles (*P* for trend < 0.05). No significant nonlinear association was found between eGDR and mortality risk (*P* for non-linearity > 0.05) (Fig. 2).

**Table 3:** Association between eGDR and mortality in the NHANES study

Variable eGDR	Event, n (%)	Model 1		Model 2		Model 3	
		HR (95%CI)	*P* value	HR (95%CI)	*P* value	HR (95%CI)	*P* value
**All-cause mortality**							
Continuous variable per unit	2046 (18.5)	0.9 (0.89 ~ 0.92)	< 0.001	0.99 (0.97 ~ 1.02)	0.613	0.94(0.92 ~ 0.97)	< 0.001
Q1(< 6.38)	873 (23.7)	1(Ref)		1(Ref)		1(Ref)	
Q2(6.38–9.14)	731 (19.8)	0.89(0.81 ~ 0.98)	0.021	1.06 (0.96 ~ 1.17)	0.234	0.94(0.85 ~ 1.05)	0.259
Q3(> 9.14)	442 (12)	0.52(0.46 ~ 0.58)	< 0.001	0.96 (0.85 ~ 1.08)	0.466	0.78(0.68 ~ 0.89)	< 0.001
Trend test	2046 (18.5)	0.74 (0.7 ~ 0.78)	< 0.001	0.99 (0.93 ~ 1.05)	0.693	0.89 (0.83 ~ 0.95)	0.001
**CVD mortality**							
Continuous variable per unit	463 (4.2)	0.84 (0.8 ~ 0.87)	< 0.001	0.92(0.88 ~ 0.96)	< 0.001	0.9(0.85 ~ 0.95)	< 0.001
Q1(< 6.38)	229 (6.2)	1(Ref)		1(Ref)		1(Ref)	
Q2(6.38–9.14)	154 (4.2)	0.72(0.58 ~ 0.88)	0.001	0.86(0.7 ~ 1.06)	0.163	0.85(0.69 ~ 1.06)	0.16
Q3(> 9.14)	80 (2.2)	0.36(0.28 ~ 0.46)	< 0.001	0.7(0.54 ~ 0.91)	0.008	0.7(0.53 ~ 0.94)	0.019
Trend test	463 (4.2)	0.62(0.55 ~ 0.69)	< 0.001	0.84(0.75 ~ 0.95)	0.007	0.84(0.73 ~ 0.97)	0.017
**Cerebrovascular disease mortality**							
Continuous variable per unit	120 (1.1)	0.88(0.82 ~ 0.95)	0.001	0.96(0.88 ~ 1.05)	0.397	0.88(0.79 ~ 0.99)	0.031
Q1(< 6.38)	58 (1.6)	1(Ref)		1(Ref)		1(Ref)	
Q2(6.38–9.14)	39 (1.1)	0.72(0.48 ~ 1.07)	0.106	0.83 (0.55 ~ 1.25)	0.374	0.67 (0.43 ~ 1.05)	0.08
Q3(> 9.14)	23 (0.6)	0.41(0.25 ~ 0.66)	< 0.001	0.76 (0.46 ~ 1.24)	0.271	0.54 (0.31 ~ 0.94)	0.03
Trend test	120 (1.1)	0.65(0.52 ~ 0.82)	< 0.001	0.86 (0.68 ~ 1.1)	0.231	0.72(0.55 ~ 0.96)	0.023

Model 1: crude. Model 2: age, sex, education, smoking status, alcohol consumption, marital status, race, and PIR. Model 3 is adjusted for model 2 covariates plus BMI, LDL-C, Cho, TG, and lipid-lowering drugs. Abbreviations: NHANES, National Health and Nutrition Examination Survey. Abbreviations: CVD, cardiovascular disease

### Stratified analysis

The association between eGDR and stroke differed across age groups (*P* for interaction < 0.05). Higher eGDR Levels exhibited differing extents of protective effects on stroke prevalence among diverse age groups, with a more pronounced impact observed in those under the age of 60. For each one-unit rise in eGDR, the prevalence of stroke reduced by 22%. For patients over 60 years old, higher eGDR still reduced the prevalence of stroke, but the reduction was only 12% (Table S2, Table S3). Conversely, no significant interactions were detected among the other subgroups (*P* for interaction > 0.05).

### Mediation analysis

We examined lipids, specifically TC, LDL-C, HDL-C, and TG, as possible mediators in the relationship between eGDR and stroke incidence and mortality (Table S4). Apart from HDL-C, which exhibited a minor mediating effect on stroke (Proportion of mediated = 9.08%, *P* = 0.044), the remaining lipid indicators did not significantly mediate the relationship between eGDR and either stroke or mortality.

### Sensitivity analyses

The results of our study demonstrated remarkable stability across multiple sensitivity analyses: excluding participants with significantly incomplete covariates (Table S5, Table S6); including individuals aged 20 or older in the NHANCE cohort (Table S7, Table S8). All these sensitivity analyses produced outcomes consistent with the primary findings.

In addition, 6,873 participants from the CHARLS study were incorporated into the second independent cohort (Figure S1). In line with the traits noted in the NHANCE population, individuals in the CHARLS cohort with higher eGDR tended to be younger, non-smokers, and non-drinkers. They exhibited higher HDL levels and lower BMI and TG concentrations (Table S10, Table S11). The prevalence of stroke in the Q3 group with higher eGDR was lower than that in the Q1 group. Subsequently, a follow-up was then carried out on 6,721 individuals without a history of stroke at baseline. Over a median of 105.4 months, 432 stroke events were recorded. In Model 3, elevated eGDR was associated with a 35% lower risk of developing stroke (HR 0.65, 95% CI 0.48–0.87, *P* = 0.001) compared to Q1 (Table S12). Stroke risk decreased significantly with higher eGDR tertiles (*P* for trend = 0.001). However, the trend of reduced all-cause mortality was not statistically significant (HR 0.93, 95% CI 0.84–1.04, *P* for trend = 0.189) (Table S12).

### Results of random-effects meta-analysis

Random-effects meta-analyses using data from both NHANES and CHARLS revealed that higher eGDR levels were significantly linked to a reduced likelihood of all-cause mortality (per 1-unit increase: pooled HR = 0.95, 95%CI:0.92–0.98, *p* = 0.001). Moderate heterogeneity was observed (*I²*=68.3%, *P* = 0.08). Trend tests indicated a stronger dose-response relationship (HR = 0.90, 95%CI:0.84–0.97, *P* = 0.006) with no heterogeneity (*I²*=0%, *P* = 0.58), consistently supporting eGDR’s protective effect across populations (Table S13).

## Discussion

In these two large-scale, nationally representative cohort studies, we identified that among non-diabetic participants, higher eGDR was significantly and negatively associated with stroke prevalence. Furthermore, elevated eGDR levels demonstrated a strong negative correlation with the risks of mortality. RCS analysis found no significant nonlinear association between eGDR and all-cause, CVD, or cerebrovascular mortality. This relationship remained significant after accounting for clinical features and other established risk factors.

IR is a common metabolic disorder in people with various diseases, including diabetes [17, 18] and obesity [19]. IR is present in approximately 20% to 25% of non-diabetic patients [18, 20] and is closely related to many cardiovascular and metabolic abnormalities, such as hypertension [21], dyslipidemia [22], NAFLD [23], and atherosclerotic cardiovascular diseases [24]. Li et al. and Sumino et al. successively discovered that IR was positively linked to the incidence and severity of silent lacunar infarcts (SLI) and served as an independent risk factor for SLI [25, 26]. The “Abnormal Glucose Regulation in Patients With Acute Stroke Across China(ACROSS-China) Study”, a registration study on IR among acute stroke patients in China, observed that individuals with IR exhibited increased risks of mortality, stroke recurrence, and adverse prognosis when compared to those without IR [27]. The prevalence of metabolic syndrome worldwide has substantially elevated the risk of stroke [28]. Improving IR is gradually becoming a new strategy for preventing or delaying the incidence of stroke [29]. The “Prospective Pioglitazone Clinical Trial in Macrovascular Events” (PROactive) Study aimed to evaluate whether the insulin sensitizer pioglitazone could reduce the incidence of macrovascular diseases. The study found that pioglitazone markedly decreased the Likelihood of recurrent stroke among high-risk individuals with type 2 diabetes [30]. The relationship between IR and stroke still requires further investigation.

The underlying pathological mechanisms linking IR to stroke are highly intricate, encompassing the accumulation of extracellular matrix deposition, inflammation, fibrosis, oxidative stress, microRNA regulation, and mitochondrial dysfunction [31–34]. All of these factors collectively contribute to vascular endothelial dysfunction and accelerate the development of atherosclerosis. Atherosclerosis is considered the primary hypothetical mechanism linking IR to ischemic stroke [35]. The brain is identified as an insulin-sensitive organ [36]. Systemic IR leading to hyperinsulinemia causes central neuronal IR, which may further inhibit cell survival by down-regulating the PI3K-AKT signaling pathway, thereby aggravating ischemic brain vascular damage. Ischemic injury induces cell death through the activation of pro-apoptotic signaling pathways [37, 38]. The underlying mechanism linking IR and stroke warrants additional exploration. Exploring pharmacological agents that enhance insulin sensitivity may represent a promising future research direction.

Previous studies have evaluated the associations between several auxiliary indicators reflecting IR and the risk of CVD, including the triglyceride-glucose (TyG) index, eGDR, and others [35, 39, 40]. Although these indices serve as independent prognostic predictors beyond traditional cardiovascular risk factors, the eGDR performs better predicting specific cardiovascular events and all-cause mortality [41–44]. Its clinical value lies in integrating key markers related to insulin resistance: Elevated HbA1c levels reflect poor long-term blood glucose control and are an essential factor leading to insulin resistance in the liver. Anthropometric measures like waist circumference indicate central obesity, a hallmark of IR. Furthermore, IR increases the risk of hypertension. All these are major drivers of cardiovascular risk. Consequently, this study selected eGDR rather than other measurement indices as the primary marker for IR. In subsequent research, we intend to conduct direct comparative studies between eGDR and other IR assessment indices in predicting cardiovascular outcomes.

Previous studies have demonstrated that elevated eGDR levels were independently and inversely linked to the risk of CVD [45]. Moreover, Han et al. found that individuals in the highest quartile of eGDR exhibited a 60% lower risk of stroke and a 30% reduction in overall mortality [12]. The China National Stroke Registry III (CNSR-III) revealed that individuals with elevated eGDR levels were more Likely to achieve favorable functional outcomes at 3 months and 1 year. These suggested that elevated eGDR levels were independent predictors of favorable and excellent functional outcomes [5, 46]. However, most of these studies focused on particular groups, especially individuals diagnosed with type 1 or type 2 diabetes [6, 47]. This could influence the understanding of the association between eGDR and stroke. Recently, several studies have pointed out that, regardless of diabetic status, eGDR was linked to a higher risk of CVD in the general population [48, 49]. In non-diabetic patients, eGDR might demonstrate greater sensitivity in forecasting CVD. The results of our research revealed that in the non-diabetic population, eGDR was negatively correlated with stroke prevalence and mortality. This was consistent with findings in diabetic populations, and these findings were extended to non-diabetic patients.

The relationship between eGDR and mortality remains a subject of ongoing debate. Many observational studies have suggested an unlined curve between eGDR levels and mortality risk [48]. At the same time, our results in the NHANES cohort indicated no nonlinear association existed between eGDR levels and all-cause, CVD, and cerebrovascular disease mortality. This was consistent with the results of Ren et al.‘s research [47]. Unlike the findings from the NHANES cohort, in the CHARLS cohort of this study, our analysis did not reveal a statistically significant link between eGDR and all-cause mortality. This suggests that, in the specific research context of the CHARLS cohort, the absence of an association between eGDR and all-cause mortality likely represents a factual negative finding. The following factors might explain the differences between the results of these two large cohorts: Differences in population characteristics and baseline eGDR distribution: CHARLS participants had a lower average age, but their average eGDR (9.5 mg/kg/min) was significantly higher than that of the NHANES cohort (7.7 mg/kg/min). This distribution difference might affect the risk prediction ability of eGDR in the CHARLS population; The impact of competing risks: Competing risks, including cancer and other age-related diseases, might dominate all-cause mortality, thereby “masking” the potential effects of eGDR on the risk of death; Unmeasured or residual confounding factors: Despite our efforts to adjust for known confounding factors, there might be differences in unmeasured factors between the cohorts. For example, the differences in the use rates of antihypertensive and lipid-lowering drugs between the two cohorts might weaken the long-term predictive value of baseline eGDR in CHARLS; Social determinants and healthcare accessibility: There were significant differences between the two cohorts in terms of socioeconomic status, healthcare accessibility, and lifestyle environments. These factors affected eGDR levels and directly influenced survival outcomes. They may interact with eGDR in different ways in different cohorts.

The negative finding in the CHARLS cohort of this study is of great value. This indicates that applying eGDR as a mortality risk prediction tool requires careful consideration of the target population’s characteristics and the context of the research. In a Chinese middle-aged and elderly population similar to CHARLS, relying solely on baseline eGDR for long-term all-cause mortality risk stratification may have limited effectiveness. Future research should focus on validating the predictive efficacy of eGDR in more diverse populations, exploring factors that affect its validity, and considering the development or validation of risk prediction models more suitable for specific populations (such as the Asian elderly).

While lipids are believed to contribute significantly to the progression of stroke [50], our research found that the influence of eGDR on outcomes was only minimally mediated by lipid levels. This suggests that there may be alternative pathways contributing to these consequences.

IR influences the incidence of stroke and mortality. This effect may grow increasingly prominent as the follow-up period extends. This study included two independent large national cohorts with relatively long follow-up periods, making our research results more universal and more helpful in clarifying the long-term prognostic value of eGDR. Waist circumference, hypertension, and HbA1c are measured in primary healthcare facilities, and the IR is comprehensively evaluated through the eGDR indicator, especially for patients without a history of diabetes. This helps formulate stroke management strategies.

Nevertheless, certain limitations should still be recognized. (1) The cross-sectional nature of NHANES data limited our ability to accurately evaluate the long-term, individual-level relationship between eGDR and stroke development over time. Although we compensated for this using CHARLS, more data are needed for verification. (2) In the present study, participants reported outcome events according to the diagnoses made by their physicians. This process could introduce memory bias and result in unavoidable misclassification. Nonetheless, this methodology is commonly recognized in cohort studies and has demonstrated a negligible effect on the overall results. (3) This study relied on a baseline assessment of eGDR, which might not entirely reflect the long-term IR and its impact on stroke progression. Future studies are required to observe the development trajectory of eGDR over a longer period.

## Conclusions

Our results indicated that among non-diabetic patients, individuals with higher eGDR (a simple indicator for measuring insulin IR) exhibited a decreased likelihood of experiencing stroke and mortality. This efficient and economical tool can be readily accessed in areas with restricted medical resources by offering easy health assessments.

## Supplementary Information

Supplementary Material 1

## Data Availability

The data derived from the National Health and Nutrition Examination Survey can be publicly accessed at https://wwwn.cde.gov/nchs/nhanes. The data derived from the China Health and Retirement Longitudinal Study can be publicly accessed at https://charls.pku.edu.cn/.

## Abbreviations

BMI: Body mass index
CHD: Coronary heart disease
CVD: Cardiovascular diseases
CHARLS: China longitudinal health and aging study
eGDR: estimated Glucose disposal rate
DBP: Diastolic pressure
FPG: Fasting plasma glucose
IR: Insulin resistance
NAFLD: Non-alcoholic fatty liver disease
NHANES: National health and nutrition examination survey
SBP: Systolic pressure
