# Supplementary material for: The association between insulin resistance assessed by estimated glucose disposal rate and stroke prevalence and mortality in non-diabetic people: evidence from two prospective cohorts
Source: Diabetol Metab Syndr. 2025 Oct 10;17:390. doi: 10.1186/s13098-025-01956-6 (PMC12512985; doi:10.1186/s13098-025-01956-6)
Supplement: Supplementary file 1 — Supplementary Material 1 [file 13098_2025_1956_MOESM1_ESM.docx]

**Catalogue**

[Figure S1. Flow Chart of participant screening in NHANES and CHARLS cohorts. 3](#_Toc26218)

[Figure S2. The multivariable-adjusted OR of stroke among the participants varied with eGDR levels. . 4](#_Toc9579)

[Table S1 Validation of the Proportional Hazards Assumption in Cox Regression Models for the Association between Variables and Mortality in NHANES Study. 5](#_Toc127)

[Figure S3. Validation of the Proportional Hazards Assumption for Cox Regression Models. 6](#_Toc25196)

[Table S2. Validation of the Proportional Hazards Assumption in Cox Regression Models for the Association between Variables and Stroke Incidence and Mortality in CHARLS Study. 8](#_Toc28893)

[Figure S4. Validation of the Proportional Hazards Assumption for Cox Regression Models. . 9](#_Toc16335)

[Table S3. Association Between eGDR and prevalence of Stroke by Specific Baseline Characteristics in NHANES Study. 11](#_Toc10762)

[Table S4. Association Between eGDR And Risk of Mortality by Specific Baseline Characteristics in NHANES Study. 13](#_Toc8893)

[Table S5. Direct and indirect effects of eGDR on the risk of Stroke and Mortality Among Participants with Stroke in NHANES Study. 15](#_Toc28734)

[Table S6. Association Between eGDR and Stroke After Including Participants with Missing Covariates in NHANES Study. 16](#_Toc23328)

[Table S7. Association Between eGDR and Mortality After Excluding Participants with Missing Covariates in NHANES Study. 17](#_Toc29112)

[Table S8. Association Between eGDR and Stroke After Including Participants Aged 20 years or older in NHANES Study. 18](#_Toc29668)

[Table S9. Association Between eGDR and Mortality After Including Participants Aged 20 years or older in NHANES Study. 19](#_Toc30841)

[Table S10. Multi-factor competing risk model compared the association between eGDR and mortality in the NHANES study. 20](#_Toc29345)

[Table S11. Baseline characteristics of 6873 participants by tertile of eGDR in CHARLS study 21](#_Toc12517)

[Table S12. Characteristics of 6721 participants by tertile of eGDR in CHARLS Study excluding with history of stroke at baseline or lost to follow-up. 23](#_Toc25234)

[Table S13. Association between eGDR and incident stroke and all-cause mortality in CHARLS study. 25](#_Toc28686)

[Table S14. Random-Effects Meta-Analysis of eGDR and All-Cause Mortality from the fully-adjusted models (Model 3). 26](#_Toc26685)

[Table S15. STROBE Checklist 27](#_Toc17720)

| NHANES | CHARLS |
| --- | --- |
| 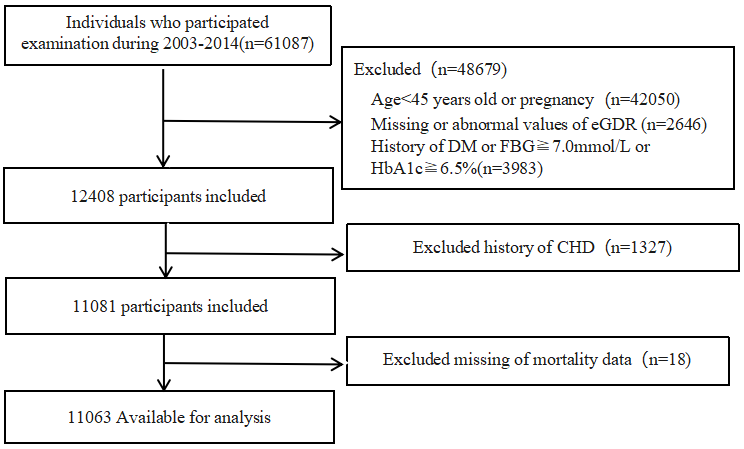 | 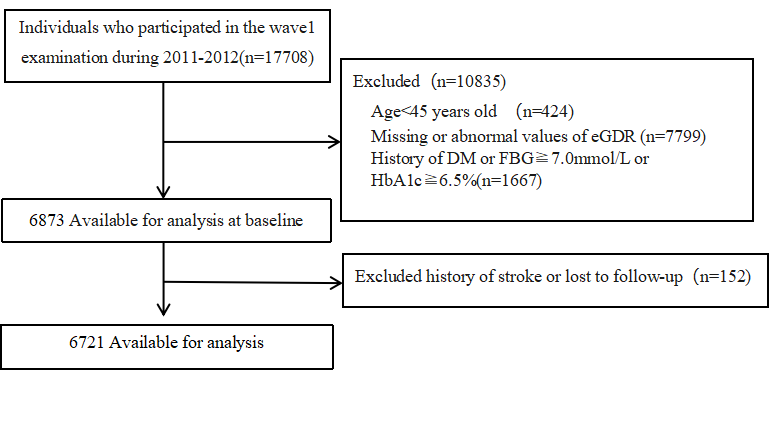 |

Figure S1. **Flow Chart of participant screening in NHANES and CHARLS cohorts.**

**
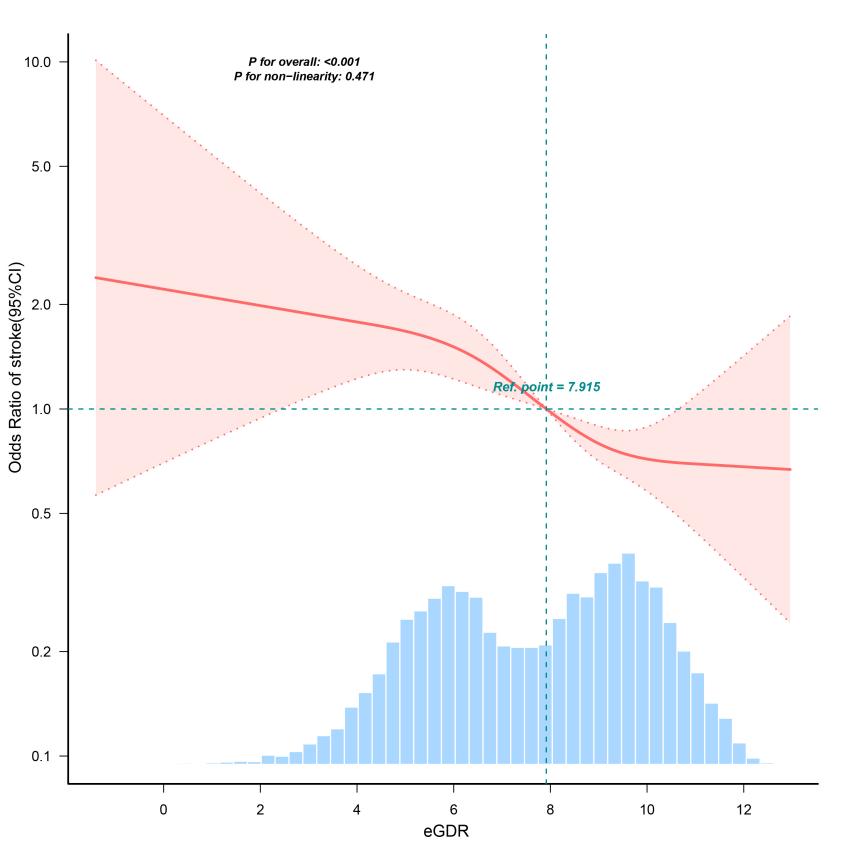
**

Figure S2. **The multivariable-adjusted OR of stroke among the participants varied with eGDR levels.** The solid line represents the multivariable-adjusted OR, and the dashed line represents the 95% CI from the RCS regression. Logistic regression analysis was adjusted for age, sex, education, smoking status, alcohol consumption, marital status, race, PIR, BMI, LDL-C, Cho, TG, and Lipid-lowering drugs.

Table S1 Validation of the Proportional Hazards Assumption in Cox Regression Models for the Association between Variables and Mortality in NHANES Study.

| Variable | **All-cause mortality** | |  | **CVD mortality** | |  | **Cerebrovascular diseases mortality** | |
| --- | --- | --- | --- | --- | --- | --- | --- | --- |
|  | Chi-sq | *P*-value |  | Chi-sq | *P*-value |  | Chi-sq | *P*-value |
| Sex | 3.308 | 0.069 |  | 0.208 | 0.649 |  | 0.205 | 0.651 |
| Age | 2.471 | 0.116 |  | 0.087 | 0.768 |  | 0.09 | 0.764 |
| PIR | 1.576 | 0.209 |  | 2.872 | 0.09 |  | 2.862 | 0.091 |
| Race | 1.615 | 0.806 |  | 4.484 | 0.344 |  | 4.485 | 0.344 |
| Education | 1.133 | 0.889 |  | 1.074 | 0.898 |  | 1.09 | 0.896 |
| Smoking status | 3.746 | 0.154 |  | 2.815 | 0.245 |  | 2.824 | 0.244 |
| Alcohol consumption | 4.143 | 0.126 |  | 2.448 | 0.118 |  | 2.451 | 0.117 |
| Marital status | 2.787 | 0.095 |  | 1.277 | 0.259 |  | 1.28 | 0.258 |
| BMI | 0.711 | 0.399 |  | 2.883 | 0.089 |  | 3.15 | 0.076 |
| eGDR | 3.214 | 0.241 |  | 0.463 | 0.793 |  | 0.458 | 0.796 |
| Cho | 0.005 | 0.946 |  | 1.22 | 0.269 |  | 1.23 | 0.267 |
| TG | 1.286 | 0.257 |  | 0.135 | 0.714 |  | 0.136 | 0.712 |
| LDL-C | 0.095 | 0.758 |  | 3.478 | 0.062 |  | 3.496 | 0.062 |
| Lipid-lowering drugs | 0.001 | 0.979 |  | 0.404 | 0.525 |  | 0.401 | 0.526 |
| GLOBAL | 28.349 | 0.257 |  | 30.624 | 0.104 |  | 30.653 | 0.103 |

| A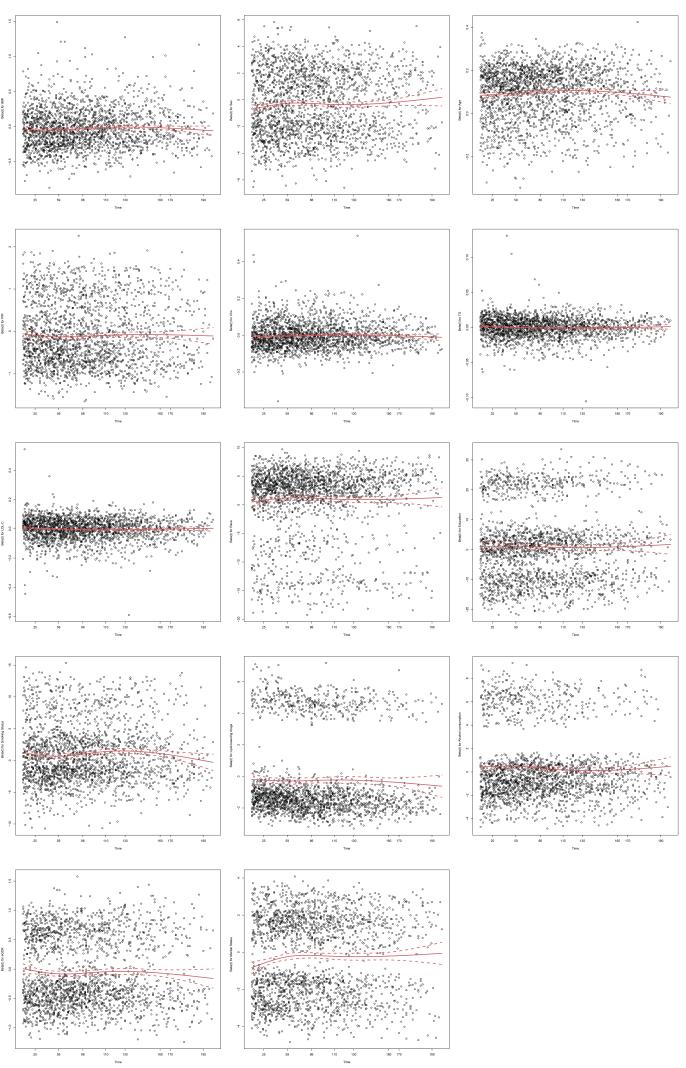 | B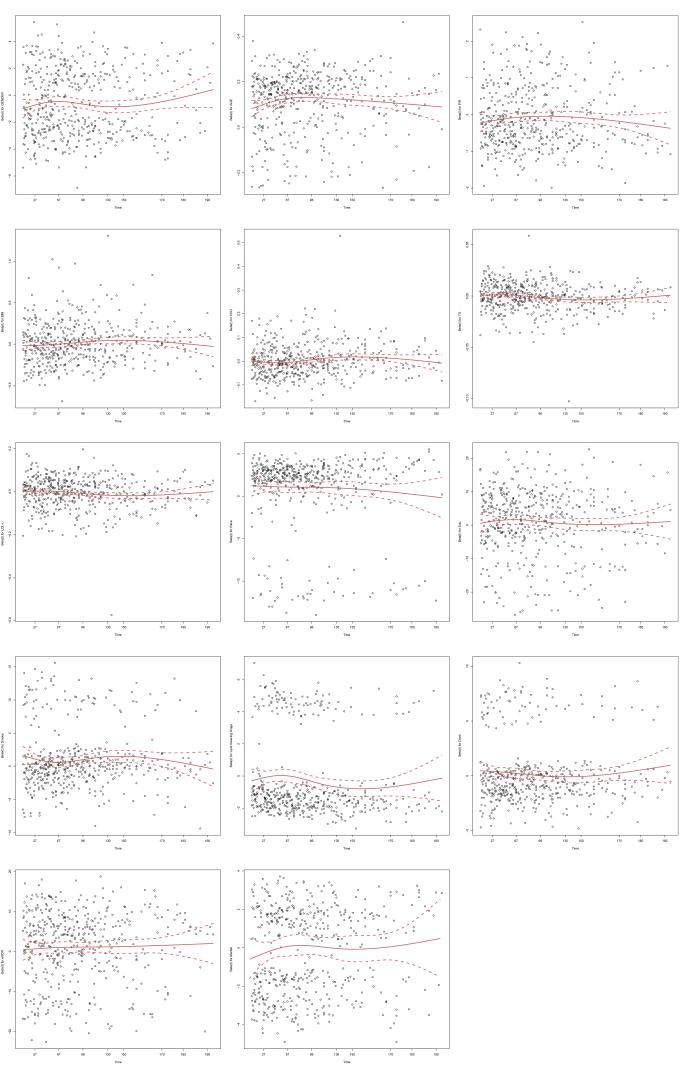 | C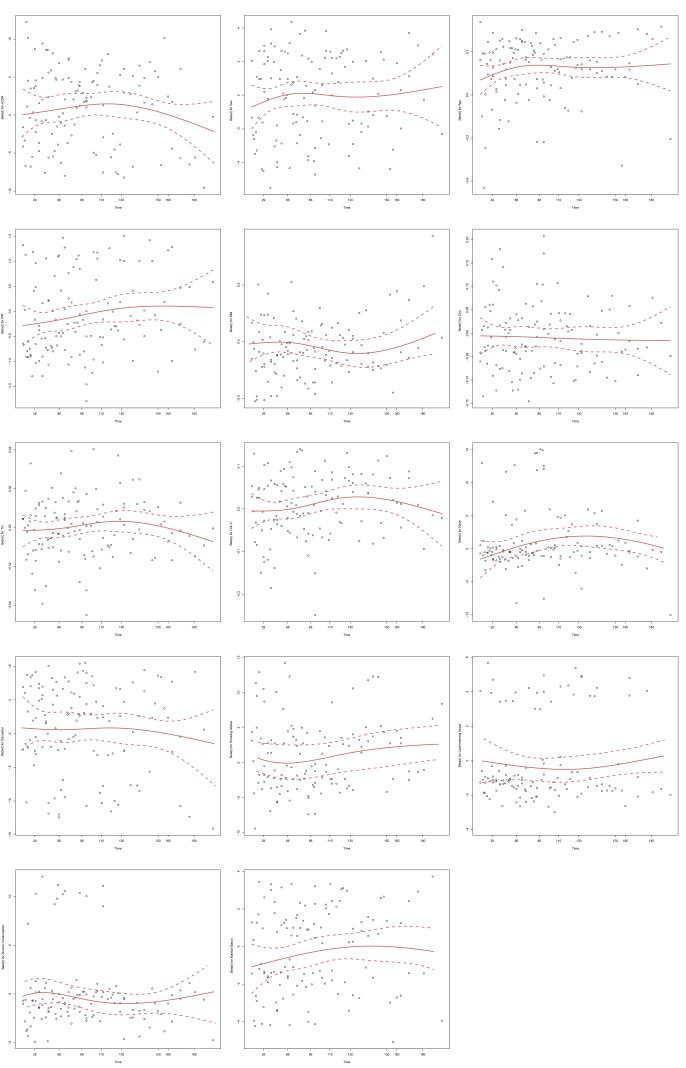 |
| --- | --- | --- |

Figure S3. **Validation of the Proportional Hazards Assumption for Cox Regression Models.** Schoenfeld residual plots assess the proportional hazards assumption for the association between key variables and mortality in the NHANES study. A global test for non-proportional hazards was performed, with a non-significant p-value (> 0.05) indicating the assumption was satisfied. Plots are shown for models predicting (A) all-cause mortality, (B) CVD mortality, and (C) cerebrovascular disease mortality. A relatively flat solid line and a non-significant p-value for each model suggest that the proportional hazards assumption was not violated.

Table S2. Validation of the Proportional Hazards Assumption in Cox Regression Models for the Association between Variables and Stroke Incidence and Mortality in CHARLS Study.

| Variable | **Stroke** | |  | **All-cause mortality** | |
| --- | --- | --- | --- | --- | --- |
|  | Chi-sq | *P*-value |  | Chi-sq | *P*-value |
| Sex | 3.606 | 0.058 |  | 0.51 | 0.475 |
| Age | 0.008 | 0.928 |  | 0.512 | 0.474 |
| Education | 1.312 | 0.519 |  | 3.182 | 0.364 |
| Smoking status | 1.251 | 0.535 |  | 1.564 | 0.458 |
| Alcohol consumption | 0.111 | 0.739 |  | 1.523 | 0.217 |
| Marital status | 0.377 | 0.539 |  | 0.018 | 0.894 |
| BMI | 0.073 | 0.787 |  | 0.003 | 0.96 |
| eGDR | 3.214 | 0.2 |  | 0.658 | 0.72 |
| CRP | 0.105 | 0.745 |  | 0.915 | 0.339 |
| UA | 1.364 | 0.243 |  | 2.823 | 0.093 |
| Cho | 0.352 | 0.553 |  | 0.102 | 0.749 |
| TG | 0.009 | 0.926 |  | 0.339 | 0.56 |
| LDL-C | 0.111 | 0.739 |  | 2.202 | 0.333 |
| lipid-lowering drugs | 2.741 | 0.098 |  | 0.063 | 0.802 |
| GLOBAL | 18.977 | 0.215 |  | 14.762 | 0.542 |

| A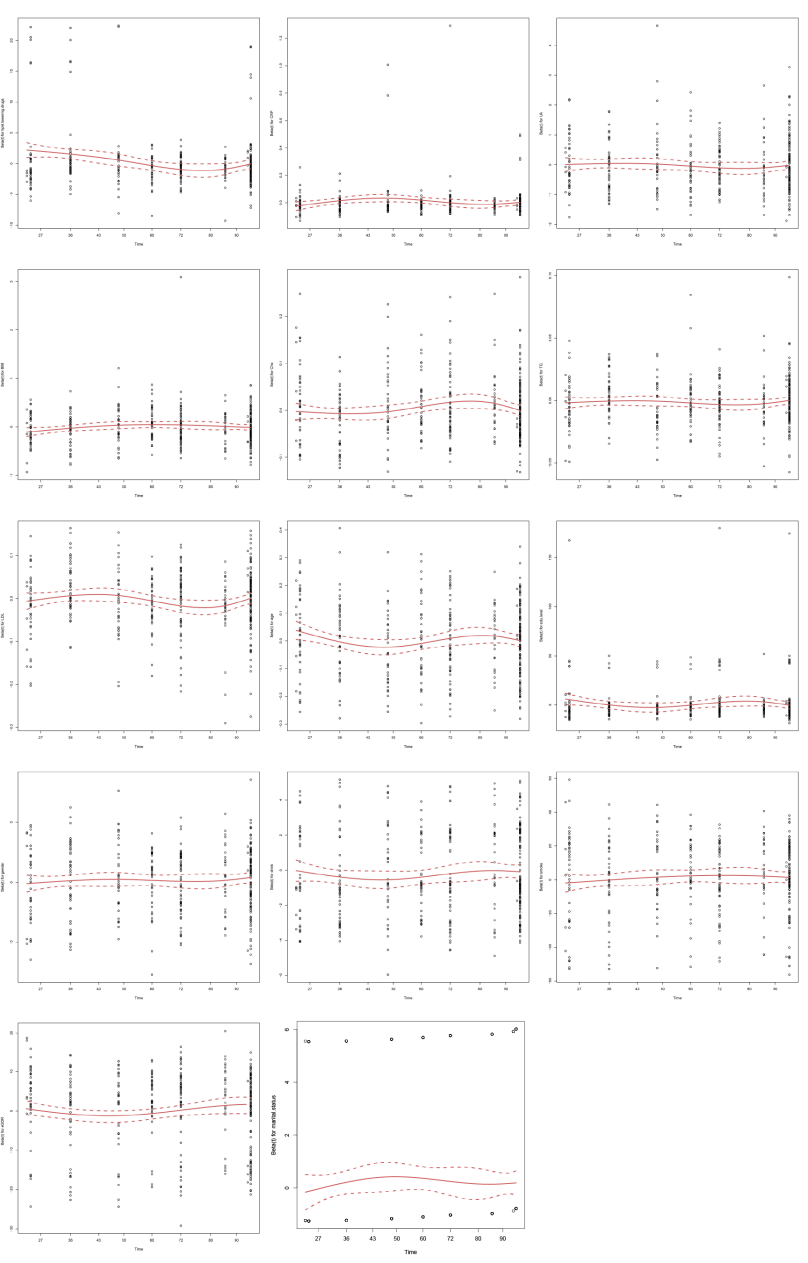 | B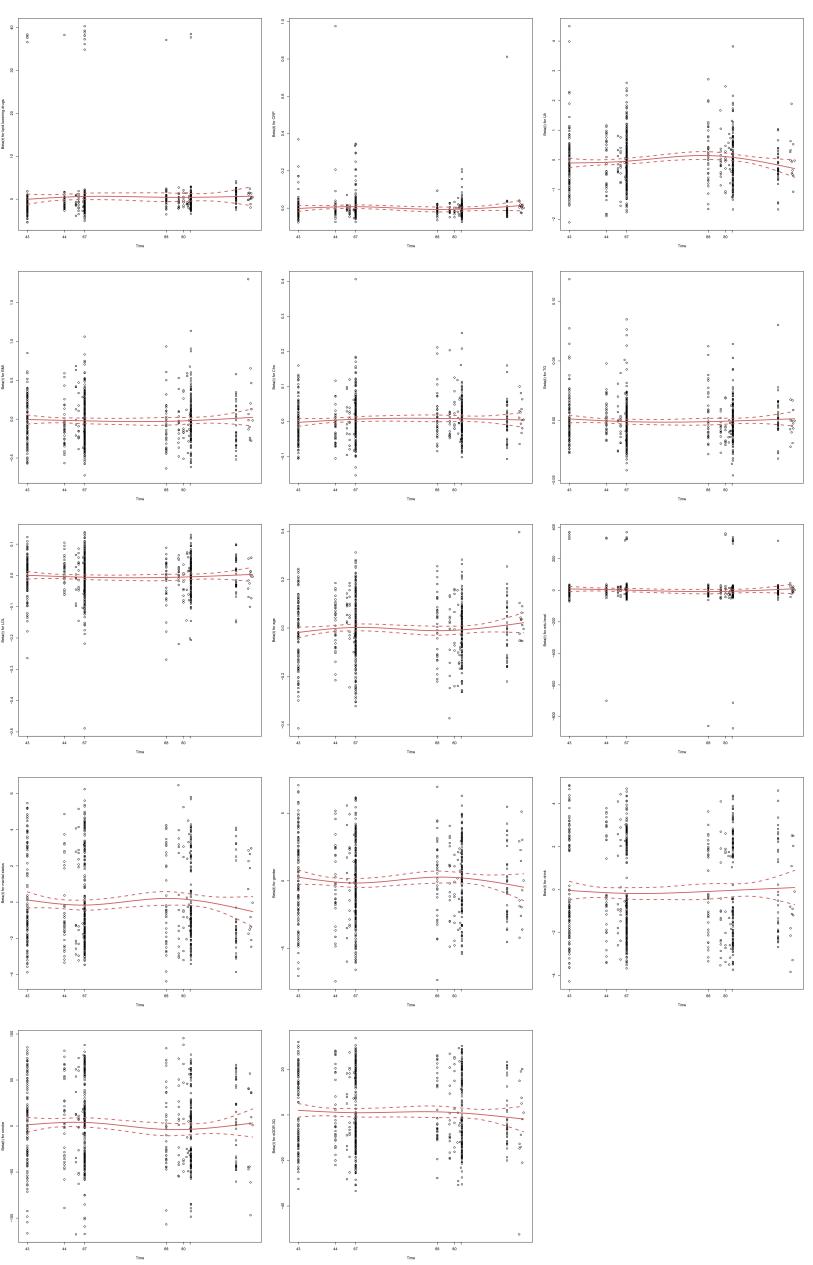 |
| --- | --- |

**Figure S4. Validation of the Proportional Hazards Assumption for Cox Regression Models.** Schoenfeld residual plots assess the proportional hazards assumption for the association between key variables and mortality in the CHARLS study. A global test for non-proportional hazards was performed, with a non-significant p-value (> 0.05) indicating the assumption was satisfied. Plots are shown for models predicting (A) Stroke Incidence, (B) All-cause mortality.

A relatively flat solid line and a non-significant p-value for each model suggest that the proportional hazards assumption was not violated.

Table S3. Association Between eGDR and prevalence of Stroke by Specific Baseline Characteristics in NHANES Study.

| Variable | Event, n (%) | Model 1 | | Model 2 | | Model 3 | | *P* for interaction |
| --- | --- | --- | --- | --- | --- | --- | --- | --- |
|  |  | OR (95%CI) | *P* value | OR (95%CI) | *P* value | OR (95%CI) | *P* value |  |
| **Sex** |  |  |  |  |  |  |  |  |
| Male | 176 (3.4) | 0.84 (0.79~0.9) | <0.001 | 0.88 (0.82~0.95) | 0.001 | 0.85 (0.78~0.94) | 0.001 | 0.454 |
| Female | 204 (3.5) | 0.82 (0.77~0.87) | <0.001 | 0.86 (0.8~0.92) | <0.001 | 0.83 (0.76~0.91) | <0.001 |  |
| **Age** |  |  |  |  |  |  |  |  |
| <60 | 91 (1.7) | 0.79 (0.73~0.86) | <0.001 | 0.8 (0.73~0.87) | <0.001 | 0.78 (0.69~0.88) | <0.001 | 0.013 |
| ≥60 | 289 (5.1) | 0.89 (0.85~0.94) | <0.001 | 0.91 (0.86~0.96) | 0.001 | 0.88 (0.81~0.94) | 0.001 |  |
| **BMI** |  |  |  |  |  |  |  |  |
| <25 | 122 (3.7) | 0.75 (0.68~0.83) | <0.001 | 0.86 (0.78~0.96) | 0.007 | 0.87 (0.78~0.98) | 0.019 | 0.926 |
| 25-30 | 138 (3.3) | 0.73 (0.66~0.8) | <0.001 | 0.85 (0.77~0.95) | 0.003 | 0.87 (0.78~0.97) | 0.011 |  |
| ≥30 | 120 (3.4) | 0.81 (0.74~0.88) | <0.001 | 0.84 (0.76~0.92) | <0.001 | 0.78 (0.69~0.88) | <0.001 |  |
| **Smoking status** | |  |  |  |  |  |  |  |
| Never | 150 (2.7) | 0.84 (0.78~0.9) | <0.001 | 0.88 (0.81~0.95) | 0.001 | 0.85 (0.76~0.94) | 0.002 | 0.263 |
| Former | 151 (4.1) | 0.85 (0.79~0.91) | <0.001 | 0.91 (0.84~0.98) | 0.019 | 0.87 (0.78~0.96) | 0.007 |  |
| Current | 79 (4.4) | 0.79 (0.71~0.87) | <0.001 | 0.81 (0.73~0.9) | <0.001 | 0.81 (0.71~0.94) | 0.005 |  |
| **Alcohol consumption** | |  |  |  |  |  |  |  |
| No | 296 (3.1) | 0.83 (0.79~0.87) | <0.001 | 0.88 (0.83~0.93) | <0.001 | 0.85 (0.79~0.92) | <0.001 | 0.987 |
| Yes | 84 (5.5) | 0.85 (0.78~0.94) | 0.001 | 0.86 (0.77~0.95) | 0.002 | 0.83 (0.73~0.96) | 0.011 |  |
| **Race** |  |  |  |  |  |  |  |  |
| Non-Hispanic White | 219 (3.8) | 0.84 (0.79~0.89) | <0.001 | 0.88 (0.82~0.94) | <0.001 | 0.84 (0.77~0.92) | <0.001 | 0.287 |
| Non-Hispanic Black | 88 (4.2) | 0.86 (0.79~0.95) | 0.002 | 0.87 (0.79~0.97) | 0.008 | 0.87 (0.75~1) | 0.049 |  |
| Hispanic | 38 (2.6) | 0.85 (0.73~0.98) | 0.026 | 0.9 (0.77~1.06) | 0.205 | 0.91 (0.75~1.12) | 0.37 |  |
| Non-Hispanic Asian | 14 (1.6) | 0.94 (0.74~1.2) | 0.631 | 0.98 (0.75~1.28) | 0.896 | 1.01 (0.71~1.42) | 0.971 |  |
| Other Race | 21 (2.7) | 0.68 (0.55~0.83) | <0.001 | 0.71 (0.56~0.88) | 0.003 | 0.55 (0.38~0.78) | 0.001 |  |

Model 1: crude. Model 2: age, sex, education, smoking status, alcohol consumption, marital status, race, and PIR. Model 3 is adjusted for model 2 covariates plus BMI, LDL-C, Cho, TG, and Lipid-lowering drugs. Abbreviations: NHANES, National Health and Nutrition Examination Survey. BMI, body mass index.

Table S4. Association Between eGDR And Risk of Mortality by Specific Baseline Characteristics in NHANES Study.

|  | **All-cause mortality*** | | | **CVD mortality*** | | | **Cerebrovascular diseases mortality*** | | |
| --- | --- | --- | --- | --- | --- | --- | --- | --- | --- |
| **Subgroup** | HR (95%CI) | *P* value | *P* for interaction | HR (95%CI) | *P* value | *P* for interaction | HR (95%CI) | *P* value | *P* for interaction |
| **Sex** |  |  |  |  |  |  |  |  |  |
| Male | 0.96 (0.92~0.99) | 0.014 | 0.553 | 0.94 (0.88~1.01) | 0.104 | 0.647 | 0.81 (0.69~0.95) | 0.011 | 0.895 |
| Female | 0.93 (0.89~0.97) | <0.001 |  | 0.85 (0.77~0.93) | <0.001 |  | 0.95 (0.81~1.11) | 0.512 |  |
| **Age** |  |  |  |  |  |  |  |  |  |
| <60 | 0.87 (0.82~0.93) | <0.001 | 0.323 | 0.76 (0.65~0.89) | 0.001 | 0.044 | 0.71 (0.48~1.06) | 0.093 | 0.881 |
| ≥60 | 0.96 (0.93~0.98) | 0.002 |  | 0.92 (0.87~0.98) | 0.006 |  | 0.9 (0.8~1.01) | 0.078 |  |
| **BMI** |  |  |  |  |  |  |  |  |  |
| <25 | 0.93 (0.89~0.97) | 0.001 | 0.252 | 0.89 (0.81~0.98) | 0.02 | 0.004 | 0.96 (0.81~1.14) | 0.653 | 0.341 |
| 25-30 | 0.94 (0.9~0.98) | 0.005 |  | 0.96 (0.88~1.06) | 0.441 |  | 0.79 (0.64~0.96) | 0.02 |  |
| ≥30 | 0.95 (0.9~1) | 0.059 |  | 0.81 (0.72~0.9) | <0.001 |  | 0.87 (0.68~1.11) | 0.267 |  |
| **Smoking status** |  |  |  |  |  |  |  |  |  |
| Never | 0.95 (0.91~0.99) | 0.012 | 0.445 | 0.91 (0.83~0.99) | 0.023 | 0.887 | 0.92 (0.78~1.09) | 0.32 | 0.485 |
| Former | 0.95 (0.91~0.99) | 0.009 |  | 0.9 (0.82~0.98) | 0.022 |  | 0.91 (0.77~1.08) | 0.277 |  |
| Current | 0.92 (0.87~0.98) | 0.007 |  | 0.88 (0.76~1.01) | 0.067 |  | 0.72 (0.51~1) | 0.05 |  |
| **Alcohol consumption** |  |  |  |  |  |  |  |  |  |
| No | 0.94 (0.92~0.97) | <0.001 | 0.128 | 0.91 (0.86~0.97) | 0.004 | 0.259 | 0.89 (0.79~1) | 0.051 | 0.753 |
| Yes | 0.93 (0.88~0.99) | 0.034 |  | 0.82 (0.71~0.96) | 0.014 |  | 0.79 (0.54~1.17) | 0.244 |  |
| **Race** |  |  |  |  |  |  |  |  |  |
| Non-Hispanic White | 0.96 (0.93~0.99) | 0.005 | 0.603 | 0.92 (0.86~0.98) | 0.016 | 0.99 | 0.94 (0.82~1.08) | 0.385 | 0.238 |
| Non-Hispanic Black | 0.93 (0.88~1) | 0.041 |  | 0.88 (0.77~1.01) | 0.079 |  | 0.55 (0.34~0.91) | 0.019 |  |
| Hispanic | 0.89 (0.81~0.97) | 0.011 |  | 0.82 (0.67~1.01) | 0.06 |  | 0.95 (0.71~1.27) | 0.734 |  |
| Non-Hispanic Asian | 0.89 (0.78~1.02) | 0.099 |  | 0.91 (0.66~1.25) | 0.564 |  | 0.23 (0.1~0.52) | <0.001 |  |
| Other Race | 0.97 (0.85~1.12) | 0.724 |  | 0.83 (0.56~1.22) | 0.34 |  | 0.97 (0.63~1.49) | 0.9 |  |

*****Adjusted for age, sex, education, smoking status, alcohol consumption, marital status, race, PIR, BMI, LDL-C, Cho, TG, and Lipid-lowering drugs. Abbreviations: NHANES, National Health and Nutrition Examination Survey. CVD, cardiovascular disease. BMI, body mass index.

Table S5. Direct and indirect effects of eGDR on the risk of Stroke and Mortality Among Participants with Stroke in NHANES Study.

| Mediators | Average Causal Mediation Effects | | | *P* value | Average Direct Effects | | | *P* value | Proportion of Mediated (%) | *P* value |
| --- | --- | --- | --- | --- | --- | --- | --- | --- | --- | --- |
|  | Estimate | Lower 95%CI | Upper 95%CI |  | Estimate | Lower 95%CI | Upper 95%CI |  |  |  |
| **Stroke*** | | | | | | | | | | |
| TG | 0.0001 | -0.0003 | 0.0006 | 0.498 | -0.0091 | -0.0152 | -0.0046 | <0.0001 | -1.53 | 0.498 |
| Cho | 0.0001 | -0.0001 | 0.0002 | 0.266 | -0.0089 | -0.0149 | -0.0046 | <0.0001 | -0.74 | 0.266 |
| LDL-C | 0.0001 | 0 | 0.0002 | 0.092 | -0.009 | -0.0151 | -0.0046 | <0.0001 | -0.82 | 0.092 |
| HDL-C | -0.0008 | -0.0018 | 0 | 0.044 | -0.0082 | -0.0142 | -0.004 | <0.0001 | 9.08 | 0.044 |
| **All-cause mortality*** | | | | | | | | | | |
| TG | 0.0001 | -0.0002 | 0.0004 | 0.634 | -0.0043 | -0.0081 | -0.001 | 0.01 | -2.23 | 0.638 |
| Cho | 0 | 0 | 0.0001 | 0.316 | -0.0043 | -0.0081 | -0.0009 | 0.008 | -0.73 | 0.324 |
| LDL-C | 0.0001 | 0 | 0.0002 | 0.1 | -0.0043 | -0.0081 | -0.001 | 0.006 | -1.6 | 0.108 |
| HDL-C | 0.0005 | -0.0002 | 0.0013 | 0.168 | -0.0047 | -0.0086 | -0.0014 | 0.008 | -12.87 | 0.172 |
| **CVD mortality*** | | | | | | | | | | |
| TG | -0.0001 | -0.0005 | 0.0002 | 0.436 | -0.0079 | -0.0143 | -0.0039 | <0.0001 | 1.6 | 0.436 |
| Cho | 0 | -0.0001 | 0 | 0.916 | -0.008 | -0.0145 | -0.0039 | <0.0001 | 0.02 | 0.916 |
| LDL-C | 0 | 0 | 0.0001 | 0.27 | -0.008 | -0.0146 | -0.0039 | <0.0001 | -0.46 | 0.27 |
| HDL-C | 0.0005 | -0.0002 | 0.0012 | 0.16 | -0.0084 | -0.0151 | -0.0043 | <0.0001 | -6.29 | 0.16 |
| **Cerebrovascular diseases** mortality***** | | | | | | | | | | |
| TG | 0.0001 | 0 | 0.0003 | 0.09 | -0.0011 | -0.0036 | 0.0002 | 0.13 | -13.44 | 0.264 |
| Cho | 0 | 0 | 0 | 0.644 | -0.001 | -0.0035 | 0.0002 | 0.166 | -0.46 | 0.71 |
| LDL-C | 0 | -0.0001 | 0 | 0.45 | -0.001 | -0.0035 | 0.0003 | 0.176 | 1.03 | 0.528 |
| HDL-C | -0.0001 | -0.0004 | 0.0002 | 0.522 | -0.0009 | -0.0034 | 0.0003 | 0.222 | 10.11 | 0.604 |

Adjusted for age, sex, education, smoking status, alcohol consumption, marital status, race, and PIR.

Table S6. Association Between eGDR and Stroke After Including Participants with Missing Covariates in NHANES Study.

| Variable | N | Event, n (%) | Model 1 | | Model 2 | | Model 3 | |
| --- | --- | --- | --- | --- | --- | --- | --- | --- |
|  |  |  | OR (95%CI) | *P* value | OR (95%CI) | *P* value | OR (95%CI) | *P* value |
| Continuous variable per unit | 4588 | 171 (3.7) | 0.84 (0.78~0.89) | <0.001 | 0.88 (0.82~0.95) | 0.001 | 0.87 (0.79~0.96) | 0.006 |
| Q1(<6.42) | 1528 | 87 (5.7) | 1(Ref) |  | 1(Ref) |  | 1(Ref) |  |
| Q2(6.42-9.18) | 1529 | 50 (3.3) | 0.56 (0.39~0.8) | 0.001 | 0.63 (0.44~0.9) | 0.012 | 0.66 (0.45~0.98) | 0.04 |
| Q3(>9.18) | 1531 | 34 (2.2) | 0.38 (0.25~0.56) | <0.001 | 0.54 (0.35~0.83) | 0.005 | 0.58 (0.36~0.95) | 0.031 |
| Trend test | 4588 | 171 (3.7) | 0.61 (0.5~0.74) | <0.001 | 0.72 (0.58~0.88) | 0.002 | 0.75 (0.59~0.96) | 0.02 |

Model 1: crude. Model 2: age, sex, education, smoking status, alcohol consumption, marital status, race, and PIR. Model 3 is adjusted for model 2 covariates plus BMI, LDL-C, Cho, TG, and Lipid-lowering drugs. Abbreviations: NHANES, National Health and Nutrition Examination Survey.

Table S7. Association Between eGDR and Mortality After Excluding Participants with Missing Covariates in NHANES Study.

| Variable | N | Event, n (%) | Model 1 | | Model 2 | | Model 3 | |
| --- | --- | --- | --- | --- | --- | --- | --- | --- |
|  |  |  | HR (95%CI) | *P* value | HR (95%CI) | *P* value | HR (95%CI) | *P* value |
| **All-cause mortality** | |  |  |  |  |  |  |  |
| Continuous variable per unit | 4588 | 847 (18.5) | 0.89 (0.86~0.92) | <0.001 | 0.98 (0.95~1.01) | 0.288 | 0.94 (0.9~0.98) | 0.003 |
| Q1(<6.42) | 1528 | 377 (24.7) | 1(Ref) |  | 1(Ref) |  | 1(Ref) |  |
| Q2(6.42-9.18) | 1529 | 287 (18.8) | 0.78 (0.67~0.91) | 0.002 | 0.95 (0.81~1.11) | 0.516 | 0.85 (0.72~1.01) | 0.058 |
| Q3(>9.18) | 1531 | 183 (12) | 0.5 (0.42~0.59) | <0.001 | 0.93 (0.77~1.12) | 0.432 | 0.78 (0.63~0.96) | 0.019 |
| Trend test | 4588 | 847 (18.5) | 0.71 (0.66~0.78) | <0.001 | 0.96 (0.88~1.05) | 0.396 | 0.88 (0.79~0.97) | 0.014 |
| **CVD mortality** |  |  |  |  |  |  |  |  |
| Continuous variable per unit | 4588 | 193 (4.2) | 0.81 (0.76~0.87) | <0.001 | 0.88 (0.82~0.95) | <0.001 | 0.88 (0.8~0.96) | 0.004 |
| Q1(<6.42) | 1528 | 99 (6.5) | 1(Ref) |  | 1(Ref) |  | 1(Ref) |  |
| Q2(6.42-9.18) | 1529 | 63 (4.1) | 0.65 (0.48~0.9) | 0.009 | 0.79 (0.58~1.09) | 0.154 | 0.83 (0.59~1.16) | 0.278 |
| Q3(>9.18) | 1531 | 31 (2) | 0.32 (0.21~0.48) | <0.001 | 0.61 (0.4~0.93) | 0.022 | 0.68 (0.42~1.08) | 0.101 |
| Trend test | 4588 | 193 (4.2) | 0.58 (0.48~0.7) | <0.001 | 0.79 (0.65~0.96) | 0.016 | 0.82 (0.66~1.03) | 0.09 |
| **Cerebrovascular diseases mortality** | | |  |  |  |  |  |  |
| Continuous variable per unit | 4588 | 51 (1.1) | 0.81 (0.72~0.91) | 0.001 | 0.87 (0.76~1.01) | 0.067 | 0.74 (0.6~0.9) | 0.002 |
| Q1(<6.42) | 1528 | 32 (2.1) | 1(Ref) |  | 1(Ref) |  | 1(Ref) |  |
| Q2(6.42-9.18) | 1529 | 12 (0.8) | 0.39 (0.2~0.75) | 0.005 | 0.49 (0.25~0.95) | 0.036 | 0.33 (0.16~0.69) | 0.003 |
| Q3(>9.18) | 1531 | 7 (0.5) | 0.22 (0.1~0.51) | <0.001 | 0.44 (0.19~1.03) | 0.057 | 0.23 (0.09~0.6) | 0.002 |
| Trend test | 4588 | 51 (1.1) | 0.45 (0.31~0.67) | <0.001 | 0.61 (0.41~0.92) | 0.018 | 0.44 (0.27~0.71) | 0.001 |

Model 1: crude. Model 2: age, sex, education, smoking status, alcohol consumption, marital status, race, and PIR. Model 3 is adjusted for model 2 covariates plus BMI, LDL-C, Cho, TG, and Lipid-lowering drugs. Abbreviations: NHANES, National Health and Nutrition Examination Survey. CVD, cardiovascular disease.

Table S8. Association Between eGDR and Stroke After Including Participants Aged 20 years or older in NHANES Study.

| Variable | N | Event, n (%) | Model 1 | | Model 1 | | Model 2 | |
| --- | --- | --- | --- | --- | --- | --- | --- | --- |
|  |  |  | OR (95%CI) | *P* value | OR (95%CI) | *P* value | OR (95%CI) | *P* value |
| Continuous variable per unit | 22330 | 426 (1.9) | 0.76 (0.73~0.79) | <0.001 | 0.87 (0.83~0.91) | <0.001 | 0.82 (0.77~0.87) | <0.001 |
| Q1(<7.82) | 7442 | 289 (3.9) | 1(Ref) |  | 1(Ref) |  | 1(Ref) |  |
| Q2(7.82-9.95) | 7443 | 88 (1.2) | 0.3 (0.23~0.38) | <0.001 | 0.54 (0.42~0.69) | <0.001 | 0.55 (0.42~0.71) | <0.001 |
| Q3(>9.9.95) | 7445 | 49 (0.7) | 0.16 (0.12~0.22) | <0.001 | 0.44 (0.32~0.61) | <0.001 | 0.41 (0.29~0.6) | <0.001 |
| Trend test | 22330 | 426 (1.9) | 0.37 (0.32~0.43) | <0.001 | 0.63 (0.54~0.73) | <0.001 | 0.61 (0.52~0.73) | <0.001 |

Model 1: crude. Model 2: age, sex, education, smoking status, alcohol consumption, marital status, race, and PIR. Model 3 is adjusted for model 2 covariates plus BMI, LDL-C, Cho, TG, and Lipid-lowering drugs. Abbreviations: NHANES, National Health and Nutrition Examination Survey.

Table S9. Association Between eGDR and Mortality After Including Participants Aged 20 years or older in NHANES Study.

| Variable | N | Event, n (%) | Model 1 | | Model 2 | | Model 3 | |
| --- | --- | --- | --- | --- | --- | --- | --- | --- |
|  |  |  | OR (95%CI) | *P* value | OR (95%CI) | *P* value | OR (95%CI) | *P* value |
| **All-cause mortality** | |  |  |  |  |  |  |  |
| Continuous variable per unit | 22330 | 2244 (10) | 0.81 (0.79~0.82) | <0.001 | 0.99 (0.97~1.01) | 0.244 | 0.94 (0.92~0.96) | <0.001 |
| Q1(<6.42) | 7442 | 1381 (18.6) | 1(Ref) |  | 1(Ref) |  | 1(Ref) |  |
| Q2(6.42-9.18) | 7443 | 527 (7.1) | 0.38 (0.34~0.42) | <0.001 | 0.85 (0.77~0.94) | 0.002 | 0.8 (0.72~0.89) | <0.001 |
| Q3(>9.18) | 7445 | 336 (4.5) | 0.23 (0.21~0.26) | <0.001 | 0.94 (0.83~1.07) | 0.346 | 0.77 (0.67~0.89) | <0.001 |
| Trend test | 22330 | 2244 (10) | 0.46 (0.43~0.49) | <0.001 | 0.94 (0.89~1) | 0.058 | 0.86 (0.8~0.92) | <0.001 |
| **CVD mortality** | |  |  |  |  |  |  |  |
| Continuous variable per unit | 22330 | 494 (2.2) | 0.75 (0.72~0.77) | <0.001 | 0.91 (0.87~0.94) | <0.001 | 0.89 (0.85~0.94) | <0.001 |
| Q1(<6.42) | 7442 | 342 (4.6) | 1(Ref) |  | 1(Ref) |  | 1(Ref) |  |
| Q2(6.42-9.18) | 7443 | 99 (1.3) | 0.29 (0.23~0.36) | <0.001 | 0.7 (0.56~0.88) | 0.002 | 0.71 (0.56~0.89) | 0.004 |
| Q3(>9.18) | 7445 | 53 (0.7) | 0.15 (0.11~0.2) | <0.001 | 0.69 (0.51~0.93) | 0.013 | 0.72 (0.52~0.99) | 0.044 |
| Trend test | 22330 | 494 (2.2) | 0.35 (0.31~0.41) | <0.001 | 0.79 (0.69~0.91) | 0.001 | 0.8 (0.69~0.93) | 0.004 |
| **Cerebrovascular diseases mortality** | |  |  |  |  |  |  |  |
| Continuous variable per unit | 22330 | 124 (0.6) | 0.78 (0.73~0.83) | <0.001 | 0.95 (0.87~1.04) | 0.25 | 0.89 (0.8~0.99) | 0.036 |
| Q1(<6.42) | 7442 | 84 (1.1) | 1(Ref) |  | 1(Ref) |  | 1(Ref) |  |
| Q2(6.42-9.18) | 7443 | 29 (0.4) | 0.34 (0.23~0.52) | <0.001 | 0.87 (0.56~1.33) | 0.513 | 0.82 (0.53~1.27) | 0.367 |
| Q3(>9.18) | 7445 | 11 (0.1) | 0.13 (0.07~0.24) | <0.001 | 0.6 (0.31~1.14) | 0.119 | 0.48 (0.24~0.96) | 0.038 |
| Trend test | 22330 | 124 (0.6) | 0.35 (0.27~0.46) | <0.001 | 0.8 (0.61~1.06) | 0.116 | 0.73 (0.55~0.98) | 0.038 |

Model 1: crude. Model 2: age, sex, education, smoking status, alcohol consumption, marital status, race, and PIR. Model 3 is adjusted for model 2 covariates plus BMI, LDL-C, Cho, TG, and lipid-lowering drugs. Abbreviations: NHANES, National Health and Nutrition Examination Survey.

Table S10. Multi-factor competing risk model compared the association between eGDR and mortality in the NHANES study.

| Variable |  | Model 1 | | Model 2 | | Model 3 | |
| --- | --- | --- | --- | --- | --- | --- | --- |
| eGDR | Event, n (%) | HR (95%CI) | *P* value | HR (95%CI) | *P* value | HR (95%CI) | *P* value |
| **All-cause mortality** | | | | | | | |
| Continuous variable per unit | 2046 (18.5) | 0.9 (0.89~0.92) | <0.001 | 0.99 (0.97~1.02) | 0.613 | 0.94(0.92~0.97) | <0.001 |
| Q1(<6.38) | 873 (23.7) | 1(Ref) |  | 1(Ref) |  | 1(Ref) |  |
| Q2(6.38-9.14) | 731 (19.8) | 0.89(0.81~0.98) | 0.021 | 1.06 (0.96~1.17) | 0.234 | 0.94(0.85~1.05) | 0.259 |
| Q3(>9.14) | 442 (12) | 0.52(0.46~0.58) | <0.001 | 0.96 (0.85~1.08) | 0.466 | 0.78(0.68~0.89) | <0.001 |
| Trend test | 2046 (18.5) | 0.74 (0.7~0.78) | <0.001 | 0.99 (0.93~1.05) | 0.693 | 0.89 (0.83~0.95) | 0.001 |
| **CVD mortality** | | | | | | | |
| Continuous variable per unit | 463 (4.2) | 0.84(0.81~0.87) | <0.001 | 0.92(0.88~0.96) | <0.001 | 0.9 (0.85~0.95) | <0.001 |
| Q1(<6.38) | 229 (6.2) | 1(Ref) |  | 1(Ref) |  | 1(Ref) |  |
| Q2(6.38-9.14) | 154 (4.2) | 0.71(0.58~0.88) | 0.001 | 0.86(0.7~1.06) | 0.163 | 0.83 (0.67~1.02) | 0.078 |
| Q3(>9.14) | 80 (2.2) | 0.37(0.29~0.48) | <0.001 | 0.7(0.54~0.91) | 0.008 | 0.7 (0.52~0.94) | 0.017 |
| Trend test | 463 (4.2) | 0.63 (0.56~0.7) | <0.001 | 0.84(0.75~0.95) | 0.007 | 0.83 (0.72~0.96) | 0.012 |
| **Cerebrovascular diseases mortality** | | | | | | | |
| Continuous variable per unit | 120 (1.1) | 0.89(0.83~0.95) | 0.001 | 0.96(0.88~1.05) | 0.397 | 0.89 (0.79~1) | 0.044 |
| Q1(<6.38) | 58 (1.6) | 1(Ref) |  | 1(Ref) |  | 1(Ref) |  |
| Q2(6.38-9.14) | 39 (1.1) | 0.72(0.48~1.08) | 0.108 | 0.83 (0.55~1.25) | 0.374 | 0.65 (0.41~1.02) | 0.061 |
| Q3(>9.14) | 23 (0.6) | 0.43 (0.27~0.7) | 0.001 | 0.76 (0.46~1.24) | 0.271 | 0.53 (0.29~0.98) | 0.042 |
| Trend test | 120 (1.1) | 0.66(0.53~0.83) | <0.001 | 0.86 (0.68~1.1) | 0.231 | 0.72 (0.53~0.98) | 0.034 |

Model 1: crude. Model 2: age, sex, education, smoking status, alcohol consumption, marital status, race, and PIR. Model 3 is adjusted for model 2 covariates plus BMI, LDL-C, Cho, TG, and lipid-lowering drugs. Abbreviations: NHANES, National Health and Nutrition Examination Survey.

Table S11. Baseline characteristics of 6873 participants by tertile of eGDR in CHARLS study

| Variables | Total (n = 6873) | eGDR(mg/kg/min) | | |  |
| --- | --- | --- | --- | --- | --- |
|  |  | Q1(<8.29)  (n = 2290) | Q2(8.29-10.91)  (n = 2292) | Q3(>10.91)  (n = 2291) | *P* value^a^ |
| Age，y | 59.0 ± 9.4 | 61.3 ± 9.6 | 57.9 ± 9.2 | 57.9 ± 9.0 | < 0.001 |
| Sex, n (%) |  |  |  |  | < 0.001 |
| Male | 3273 (47.6) | 1045 (45.6) | 1063 (46.4) | 1165 (50.9) |  |
| Female | 3600 (52.4) | 1245 (54.4) | 1229 (53.6) | 1126 (49.1) |  |
| Education, n (%) |  |  |  |  | 0.02 |
| Less than 9th grade | 6228 (90.6) | 2104 (91.9) | 2053 (89.6) | 2071 (90.4) |  |
| 9-11th grade | 561 (8.2) | 159 (6.9) | 212 (9.2) | 190 (8.3) |  |
| High school | 70 (1.0) | 22 (1) | 26 (1.1) | 22 (1) |  |
| College or above | 14 (0.2) | 5 (0.2) | 1 (0) | 8 (0.3) |  |
| Smoking status, n (%) |  |  |  |  | < 0.001 |
| Never | 4116 (59.9) | 1406 (61.4) | 1423 (62.1) | 1287 (56.2) |  |
| Former | 586 (8.5) | 213 (9.3) | 221 (9.6) | 152 (6.6) |  |
| Current | 2171 (31.6) | 671 (29.3) | 648 (28.3) | 852 (37.2) |  |
| Alcohol consumption, n (%) | |  |  |  | 0.039 |
| No | 4531 (65.9) | 1549 (67.6) | 1514 (66.1) | 1468 (64.1) |  |
| Yes | 2342 (34.1) | 741 (32.4) | 778 (33.9) | 823 (35.9) |  |
| Marital status, n (%) |  |  |  |  | < 0.001 |
| Not married nor living with a partner | 5765 (83.9) | 1856 (81) | 1955 (85.3) | 1954 (85.3) |  |
| Married or living with a partner | 1108 (16.1) | 434 (19) | 337 (14.7) | 337 (14.7) |  |
| BMI, kg/m2 | 372.4 ± 9269.1 | 537.4 ± 11123.2 | 275.4 ± 8501.7 | 304.7 ± 7861.2 | 0.577 |
| HbA1c, % | 5.1 ± 0.4 | 5.1 ± 0.4 | 5.1 ± 0.4 | 5.0 ± 0.4 | < 0.001 |
| FBG, mmol/L | 99.9 ± 11.9 | 101.6 ± 11.9 | 100.3 ± 11.7 | 97.9 ± 11.7 | < 0.001 |
| Cho, mg/dl | 192.1 ± 37.3 | 196.7 ± 38.1 | 193.2 ± 36.8 | 186.5 ± 36.3 | < 0.001 |
| HDL, mg/dl | 52.3 ± 15.1 | 49.9 ± 14.3 | 50.8 ± 14.5 | 56.2 ± 15.5 | < 0.001 |
| LDL, mg/dl | 116.7 ± 33.9 | 120.2 ± 35.4 | 118.2 ± 33.4 | 111.7 ± 32.3 | < 0.001 |
| TG, mg/dl | 100.0 (72.6, 144.3) | 113.3 (80.5, 162.0) | 105.3 (74.3, 147.8) | 85.8 (63.7, 119.9) | < 0.001 |
| eGDR, mg/kg/min | 9.5 ± 2.1 | 6.9 ± 0.8 | 10.0 ± 0.7 | 11.6 ± 0.5 | < 0.001 |
| hs-CRP, mg/L | 2.6 ± 7.4 | 3.0 ± 8.4 | 2.3 ± 5.4 | 2.4 ± 8.2 | < 0.001 |
| UA, mg/dL | 4.4 ± 1.2 | 4.7 ± 1.3 | 4.4 ± 1.2 | 4.2 ± 1.2 | < 0.001 |
| SBP, mmHg | 129.9 ± 21.1 | 148.7 ± 20.0 | 123.7 ± 15.8 | 117.2 ± 11.3 | < 0.001 |
| DBP, mmHg | 75.6 ± 11.9 | 84.2 ± 12.0 | 73.2 ± 9.7 | 69.4 ± 8.4 | < 0.001 |
| Hypertension, n (%) |  |  |  |  | < 0.001 |
| No | 4312 (62.7) | 13 (0.6) | 2009 (87.7) | 2290 (100) |  |
| Yes | 2561 (37.3) | 2277 (99.4) | 283 (12.3) | 1 (0) |  |
| Antihypertensive drugs, n (%) | |  |  |  | < 0.001 |
| No | 5845 (85.0) | 1340 (58.5) | 2214 (96.6) | 2291 (100) |  |
| Yes | 1028 (15.0) | 950 (41.5) | 78 (3.4) | 0 (0) |  |
| Hyperlipemia, n (%) | |  |  |  | < 0.001 |
| No | 3927 (57.1) | 1073 (46.9) | 1240 (54.1) | 1614 (70.4) |  |
| Yes | 2946 (42.9) | 1217 (53.1) | 1052 (45.9) | 677 (29.6) |  |
| Lipid-lowering drugs, n (%) |  |  |  |  | < 0.001 |
| No | 6661 (96.9) | 2153 (94) | 2238 (97.6) | 2270 (99.1) |  |
| Yes | 212 (3.1) | 137 (6) | 54 (2.4) | 21 (0.9) |  |
| Stroke, n (%) |  |  |  |  | < 0.001 |
| No | 6753 (98.3) | 2221 (97) | 2260 (98.6) | 2272 (99.2) |  |
| Yes | 120 (1.7) | 69 (3) | 32 (1.4) | 19 (0.8) |  |

Values for categorical variables were presented as count (%), mean ± SD. TG variable were presented as median and interquartile range (IQR). Abbreviations: BMI, body mass index; Cho, Cholesterol; TG, triglyceride; hs-CRP: high-sensitivity C-reactive protein; DBP, diastolic blood pressure; eGDR, estimated glucose disposal rate; FBG, Fasting blood glucose; HbA1c, Hemoglobin A1c; HDL-C, high-density lipoprotein cholesterol; LDL-C, low-density lipoprotein cholesterol; SBP, systolic blood pressure; UA, uric acid.

^a^ *P* values derived from χ2 tests (categorical variables) or ANOVA (continuous variables) comparing values across tertiles.

Table S12. Characteristics of 6721 participants by tertile of eGDR in CHARLS Study excluding with history of stroke at baseline or lost to follow-up.

| Variables | Total (n = 6721) |  | eGDR (mg/kg/min) |  |  |
| --- | --- | --- | --- | --- | --- |
|  |  | Q1(<8.30)  (n = 2236) | Q2(8.30-10.91)  (n = 2244) | Q3(>10.91)  (n = 2241) | *P* value ^a^ |
| Age，y | 58.9 ± 9.4 | 61.2 ± 9.6 | 57.8 ± 9.2 | 57.7 ± 9.0 | < 0.001 |
| Sex, n (%) |  |  |  |  | < 0.001 |
| Male | 3211 (47.8) | 1024 (45.8) | 1039 (46.3) | 1148 (51.2) |  |
| Female | 3510 (52.2) | 1212 (54.2) | 1205 (53.7) | 1093 (48.8) |  |
| Education, n (%) |  |  |  |  | 0.03 |
| Less than 9th grade | 6083 (90.5) | 2051 (91.7) | 2008 (89.5) | 2024 (90.3) |  |
| 9-11th grade | 555 (8.3) | 159 (7.1) | 208 (9.3) | 188 (8.4) |  |
| High school | 69 (1.0) | 21 (0.9) | 27 (1.2) | 21 (0.9) |  |
| College or above | 14 (0.2) | 5 (0.2) | 1 (0) | 8 (0.4) |  |
| Smoking status, n (%) |  |  |  |  | < 0.001 |
| Never | 4020 (59.8) | 1367 (61.1) | 1398 (62.3) | 1255 (56) |  |
| Former | 561 (8.3) | 197 (8.8) | 217 (9.7) | 147 (6.6) |  |
| Current | 2140 (31.8) | 672 (30.1) | 629 (28) | 839 (37.4) |  |
| Alcohol consumption, n (%) |  |  |  |  | 0.044 |
| No | 4421 (65.8) | 1506 (67.4) | 1484 (66.1) | 1431 (63.9) |  |
| Yes | 2300 (34.2) | 730 (32.6) | 760 (33.9) | 810 (36.1) |  |
| Marital status, n (%) |  |  |  |  | < 0.001 |
| Married or living  with a partner | 5648 (84.0) | 1817 (81.3) | 1917 (85.4) | 1914 (85.4) |  |
| Not married nor  living with a partner | 1073 (16.0) | 419 (18.7) | 327 (14.6) | 327 (14.6) |  |
| BMI, kg/m2 | 23.2 ± 3.7 | 24.7 ± 3.8 | 24.2 ± 3.3 | 20.8 ± 2.5 | < 0.001 |
| HbA1c, % | 5.1 ± 0.4 | 5.1 ± 0.4 | 5.1 ± 0.4 | 5.0 ± 0.4 | < 0.001 |
| FBG, mmol/L | 5.6 ± 0.7 | 5.6 ± 0.7 | 5.6 ± 0.6 | 5.4 ± 0.7 | < 0.001 |
| Cho, mg/dL | 192.3 ± 37.4 | 197.0 ± 38.2 | 193.3 ± 36.8 | 186.6 ± 36.4 |  |
| HDL, mg/dL | 52.4 ± 15.0 | 50.2 ± 14.4 | 50.8 ± 14.5 | 56.1 ± 15.4 |  |
| LDL, mg/dL | 116.9 ± 33.9 | 120.6 ± 35.4 | 118.3 ± 33.3 | 112.0 ± 32.3 |  |
| TG, mg/dL | 100.0 (72.6, 144.3) | 112.4 (80.5, 161.3) | 104.9 (75.0, 147.8) | 85.8 (63.7, 119.5) |  |
| hs-CRP, mg/L | 2.6 ± 7.7 | 3.0 ± 8.2 | 2.2 ± 5.4 | 2.5 ± 9.0 | 0.004 |
| UA, mg/dL | 4.4 ± 1.2 | 4.7 ± 1.3 | 4.4 ± 1.2 | 4.2 ± 1.2 | < 0.001 |
| eGDR, mg/kg/min | 9.5 ± 2.1 | 6.9 ± 0.8 | 10.0 ± 0.7 | 11.6 ± 0.5 | < 0.001 |
| SBP, mmHg | 129.9 ± 21.1 | 149.0 ± 20.0 | 123.5 ± 15.8 | 117.2 ± 11.3 | < 0.001 |
| DBP, mmHg | 75.6 ± 12.0 | 84.4 ± 12.0 | 73.1 ± 9.7 | 69.4 ± 8.4 | < 0.001 |
| Hypertension, n (%) |  |  |  |  | < 0.001 |
| No | 4237 (63.0) | 12 (0.5) | 1984 (88.4) | 2241 (100) |  |
| Yes | 2484 (37.0) | 2224 (99.5) | 260 (11.6) | 0 (0) |  |
| Antihypertensive drugs, n (%) |  |  |  |  | < 0.001 |
| No | 5738 (85.4) | 1324 (59.2) | 2173 (96.8) | 2241 (100) |  |
| Yes | 983 (14.6) | 912 (40.8) | 71 (3.2) | 0 (0) |  |
| Hyperlipemia, n (%) |  |  |  |  | < 0.001 |
| No | 3849 (57.3) | 1055 (47.2) | 1218 (54.3) | 1576 (70.3) |  |
| Yes | 2872 (42.7) | 1181 (52.8) | 1026 (45.7) | 665 (29.7) |  |
| Lipid-lowering drugs, n (%) |  |  |  |  | < 0.001 |
| No | 6515 (96.9) | 2104 (94.1) | 2190 (97.6) | 2221 (99.1) |  |
| Yes | 206 (3.1) | 132 (5.9) | 54 (2.4) | 20 (0.9) |  |
| Stroke new, n (%) |  |  |  |  | < 0.001 |
| No | 6289 (93.6) | 2019 (90.3) | 2117 (94.3) | 2153 (96.1) |  |
| Yes | 432 (6.4) | 217 (9.7) | 127 (5.7) | 88 (3.9) |  |

Values for categorical variables were presented as count (%), mean ± SD. TG variable were presented as median and interquartile range (IQR). Abbreviations: BMI, body mass index; Cho, Cholesterol; TG, triglyceride; hs-CRP: high-sensitivity C-reactive protein; DBP, diastolic blood pressure; eGDR, estimated glucose disposal rate; FBG, Fasting blood glucose; HbA1c, Hemoglobin A1c; HDL-C, high-density lipoprotein cholesterol; LDL-C, low-density lipoprotein cholesterol; SBP, systolic blood pressure; UA, uric acid.

^a^ *P* values derived from χ2 tests (categorical variables) or ANOVA (continuous variables) comparing values across tertiles.

Table S13. Association between eGDR and incident stroke and all-cause mortality in CHARLS study.

| Variable | Event, n (%) | Model 1 | | Model 2 | | Model 3 | |
| --- | --- | --- | --- | --- | --- | --- | --- |
|  |  | HR (95%CI) | *P* value | HR (95%CI) | *P* value | HR (95%CI) | *P* value |
| **Stroke** |  |  |  |  |  |  |  |
| Continuous variable per unit | 432 (6.4) | 0.94 (0.89~0.98) | 0.005 | 0.93 (0.89~0.98) | 0.005 | 0.92 (0.87~0.97) | 0.004 |
| Q1(<8.30) | 217 (9.7) | 1(Ref) |  | 1(Ref) |  | 1(Ref) |  |
| Q2(8.30-10.91) | 127 (5.7) | 0.75(0.6~0.93) | 0.009 | 0.73 (0.58~0.91) | 0.006 | 0.72 (0.57~0.91) | 0.006 |
| Q3(>10.91) | 88 (3.9) | 0.69 (0.54~0.89) | 0.004 | 0.69 (0.53~0.89) | 0.004 | 0.65 (0.48~0.87) | 0.004 |
| Trend test | 432 (6.4) | 0.82 (0.72~0.92) | 0.001 | 0.81 (0.72~0.92) | 0.002 | 0.79 (0.68~0.91) | 0.001 |
| **All-cause mortality** | |  |  |  |  |  |  |
| Continuous variable per unit | 669 (10) | 0.99 (0.95~1.02) | 0.511 | 0.99 (0.96~1.03) | 0.612 | 0.98 (0.94~1.02) | 0.282 |
| Q1(<8.30) | 286 (12.8) | 1(Ref) |  | 1(Ref) |  | 1(Ref) |  |
| Q2(8.30-10.91) | 185 (8.2) | 0.86 (0.72~1.04) | 0.125 | 0.88 (0.73~1.06) | 0.185 | 0.86 (0.71~1.05) | 0.133 |
| Q3(>10.91) | 198 (8.8) | 0.93 (0.77~1.11) | 0.406 | 0.93 (0.78~1.12) | 0.466 | 0.88 (0.71~1.08) | 0.215 |
| Trend test | 669 (10) | 0.96 (0.87~1.05) | 0.344 | 0.96 (0.88~1.06) | 0.41 | 0.93 (0.84~1.04) | 0.189 |

Model 1: crude. Model 2: age, sex, education, smoking status, alcohol consumption, marital status. Model 3 is adjusted for model 2 covariates plus BMI, LDL-C, Cho, TG, Lipid-lowering drugs, hs-CRP, UA.

Table S14. Random-Effects Meta-Analysis of eGDR and All-Cause Mortality from the fully-adjusted models (Model 3).

| Analysis Type | Study | HR (95% CI) | *P*-value | Pooled HR (95% CI) | *P*-pooled | *I²*(%) | *P*-heterogeneity |
| --- | --- | --- | --- | --- | --- | --- | --- |
| Continuous variable per unit | NHANES | 0.94 (0.92-0.97) | <0.001 | 0.95 (0.92-0.98) | 0.001 | 68.3 | 0.08 |
|  | CHARLS | 0.98 (0.94-1.02) | 0.282 |  |  |  |  |
| Trend Test | NHANES | 0.89 (0.83-0.95) | 0.001 | 0.90 (0.84-0.97) | 0.006 | 0 | 0.58 |
|  | CHARLS | 0.93 (0.84-1.04) | 0.189 |  |  |  |  |

Model 3 is adjusted for age, sex, education, smoking status, alcohol consumption, marital status, race (NHANES only), PIR (NHANES only), BMI, LDL-C, Cho, TG, Lipid-lowering drugs, hs-CRP (CHARLS only), and UA (CHARLS only).

Table S15. STROBE Checklist

|  | Item No | Recommendation | Page /Paragraph |
| --- | --- | --- | --- |
| **Title and abstract** | 1 | (*a*) Indicate the study’s design with a commonly used term in the title or the abstract | Page 1. Title: Evidence from Two Prospective Cohort Studies |
|  |  | (*b*) Provide in the abstract an informative and balanced summary of what was done and what was found | Page1-3. Abstract includes background, methods, results, and conclusions. |
| Introduction | | |  |
| Background/rationale | 2 | Explain the scientific background and rationale for the investigation being reported | Page3-4. Background includes the burden of stroke, the association between IR and CVD, and the advantages of eGDR. |
| Objectives | 3 | State specific objectives, including any prespecified hypotheses | Page 4. The final paragraph of the Background section: "To address this ..." |
| Methods | | |  |
| Study design | 4 | Present key elements of study design early in the paper | Page 4-5. Methods："prospective cohorts" |
| Setting | 5 | Describe the setting, locations, and relevant dates, including periods of recruitment, exposure, follow-up, and data collection | Page 4-6. Methods："NHANES (2003-2014)"; "CHARLS (2011-2012)". Mortality assessment section. |
| Participants | 6 | (*a*) Give the eligibility criteria, and the sources and methods of selection of participants | Page 4-5. Methods：Participant Cohort |
| Variables | 7 | Clearly define all outcomes, exposures, predictors, potential confounders, and effect modifiers. Give diagnostic criteria, if applicable | Page 5-6. Methods：Determination of Exposure Factors and Outcome Measures;Mortality assessment;Covariates of interest |
| Data sources/ measurement | 8* | For each variable of interest, give sources of data and details of methods of assessment (measurement). Describe comparability of assessment methods if there is more than one group | Page4-6. Methods："Questionnaire, physical examination, and laboratory tests" |
| Bias | 9 | Describe any efforts to address potential sources of bias | Page 7-8. Methods: "Multivariate adjustment, sensitivity analysis (excluding missing/ expanding age range)" |
| Study size | 10 | Explain how the study size was arrived at | Page 4. Methods：Participant Cohort |
| Quantitative variables | 11 | Explain how quantitative variables were handled in the analyses. If applicable, describe which groupings were chosen and why | Page 7. Method: Continuous variable of eGDR and tertiles |
| Statistical methods | 12 | (*a*) Describe all statistical methods, including those used to control for confounding | Page 7. Method: Clearly list Cox/logistic models, RCS, Fine-Gray models, mediation analysis, subgroup/interaction analysis, and missing data handling. |
|  |  | (*b*) Describe any methods used to examine subgroups and interactions | Page 8. Method: Stratified by age/gender, etc. |
|  |  | (*c*) Explain how missing data were addressed | Page 8. Method: "Deletion Method + Multiple Imputation" |
|  |  | (*d*) If applicable, describe analytical methods taking account of sampling strategy | Not Applicable |
|  |  | (*e*) Describe any sensitivity analyses | Page 8. Method: Statistical Analysis |
| Results | | |  |
| Participants | 13* | (a) Report numbers of individuals at each stage of study—eg numbers potentially eligible, examined for eligibility, confirmed eligible, included in the study, completing follow-up, and analysed | Page 9. Results: Baseline features |
|  |  | (b) Give reasons for non-participation at each stage | Refer to "Fig. S1" |
|  |  | (c) Consider use of a flow diagram | Refer to "Fig. S1" |
| Descriptive data | 14* | (a) Give characteristics of study participants (eg demographic, clinical, social) and information on exposures and potential confounders | Table 1（NHANES）、Table S10-11（CHARLS） |
|  |  | (b) Indicate number of participants with missing data for each variable of interest | Refer to "Fig. S1" |
| Outcome data | 15* | Report numbers of outcome events or summary measures | Page 9,10,12. Results. "380 cases of stroke"; "2,046 deaths"; "432 cases of stroke in CHARLS" |
| Main results | 16 | (*a*) Give unadjusted estimates and, if applicable, confounder-adjusted estimates and their precision (eg, 95% confidence interval). Make clear which confounders were adjusted for and why they were included | Table 2-3；Table S12（CHARLS） |
|  |  | (*b*) Report category boundaries when continuous variables were categorized | Table 2-3；Table S12（CHARLS） |
|  |  | (*c*) If relevant, consider translating estimates of relative risk into absolute risk for a meaningful time period | Not Applicable |
| Other analyses | 17 | Report other analyses done—eg analyses of subgroups and interactions, and sensitivity analyses | Subgroup (Tables S2 - S3), mediation (Table S4), sensitivity (Tables S5 - S8), and meta-analysis (Table S13) |
| Discussion | | |  |
| Key results | 18 | Summarise key results with reference to study objectives | Page13.Discussion: The first paragraph |
| Limitations | 19 | Discuss limitations of the study, taking into account sources of potential bias or imprecision. Discuss both direction and magnitude of any potential bias | Page13. The Discussion section concludes by listing three limitations. |
| Interpretation | 20 | Give a cautious overall interpretation of results considering objectives, limitations, multiplicity of analyses, results from similar studies, and other relevant evidence | Discussion: Comparing literature and exploring mechanisms |
| Generalisability | 21 | Discuss the generalisability (external validity) of the study results | Discussion: Analysis of Differences between China and the United States and Applicability Limitations |
| Other information | | |  |
| Funding | 22 | Give the source of funding and the role of the funders for the present study and, if applicable, for the original study on which the present article is based | Declaration: "No funding" |

*Give information separately for exposed and unexposed groups.
